# The Carcinogenic Liver Fluke, *Clonorchis sinensis*: New Assembly, Reannotation and Analysis of the Genome and Characterization of Tissue Transcriptomes

**DOI:** 10.1371/journal.pone.0054732

**Published:** 2013-01-30

**Authors:** Yan Huang, Wenjun Chen, Xiaoyun Wang, Hailiang Liu, Yangyi Chen, Lei Guo, Fang Luo, Jiufeng Sun, Qiang Mao, Pei Liang, Zhizhi Xie, Chenhui Zhou, Yanli Tian, Xiaoli Lv, Lisi Huang, Juanjuan Zhou, Yue Hu, Ran Li, Fan Zhang, Huali Lei, Wenfang Li, Xuchu Hu, Chi Liang, Jin Xu, Xuerong Li, Xinbing Yu

**Affiliations:** 1 Department of Parasitology, Zhongshan School of Medicine, Sun Yat-sen University, Guangzhou, Guangdong, People’s Republic of China; 2 Key Laboratory for Tropical Diseases Control of Ministry of Education, Sun Yat-sen University, Guangzhou, Guangdong, People’s Republic of China; 3 Guangzhou iGenomics Co., Ltd, Guangzhou, Guangdong, People’s Republic of China; University of Melbourne, Australia

## Abstract

*Clonorchis sinensis (C. sinensis)*, an important food-borne parasite that inhabits the intrahepatic bile duct and causes clonorchiasis, is of interest to both the public health field and the scientific research community. To learn more about the migration, parasitism and pathogenesis of *C. sinensis* at the molecular level, the present study developed an upgraded genomic assembly and annotation by sequencing paired-end and mate-paired libraries. We also performed transcriptome sequence analyses on multiple *C. sinensis* tissues (sucker, muscle, ovary and testis). Genes encoding molecules involved in responses to stimuli and muscle-related development were abundantly expressed in the oral sucker. Compared with other species, genes encoding molecules that facilitate the recognition and transport of cholesterol were observed in high copy numbers in the genome and were highly expressed in the oral sucker. Genes encoding transporters for fatty acids, glucose, amino acids and oxygen were also highly expressed, along with other molecules involved in metabolizing these substrates. All genes involved in energy metabolism pathways, including the β-oxidation of fatty acids, the citrate cycle, oxidative phosphorylation, and fumarate reduction, were expressed in the adults. Finally, we also provide valuable insights into the mechanism underlying the process of pathogenesis by characterizing the secretome of *C. sinensis*. The characterization and elaborate analysis of the upgraded genome and the tissue transcriptomes not only form a detailed and fundamental *C. sinensis* resource but also provide novel insights into the physiology and pathogenesis of *C. sinensis*. We anticipate that this work will aid the development of innovative strategies for the prevention and control of clonorchiasis.

## Introduction

*Clonorchis sinensis* (*C. sinensis*) has been proven to be the causative agent of clonorchiasis, which is endemic in China, Korea and Vietnam [1], [2], [3]. As an important food-borne parasite, *C. sinensis* has afflicted more than 35 million people in Asia and approximately 15 million in China, creating a socio-economic burden in epidemic regions [4]. Most clonorchiasis cases are due to the consumption of raw freshwater fish containing infective *C. sinensis* metacercariae, which excyst in the duodenum until they grow into juvenile *C. sinensis* and then migrate into the bile ducts of their host [5], [6]. Adult worms rely on bile juice for growth, reproduction and egg-laying. Both experimental and epidemiological evidence have implied that long-term infections by liver flukes lead to chronic pathological changes, including cholangitis, cholecystitis, cholelithiasis, cholangiectasis, adenomatous hyperplasia, hepatomegaly, hepatic fibrosis and cholangiocarcinoma (CCA), a malignant bile duct tumor found in intrahepatic or extrahepatic biliary trees [7], [8], [9]. Although many agents can be responsible for cholangiocarcinoma, liver flukes, particularly *C. sinensis* and *Opisthorchis viverrini* (*O. viverrini*), have been shown to have the strongest risk factors [10]. Furthermore, *C. sinensis* was recently classified along with *O. viverrini* as a Group I biological carcinogen by the World Health Organization [11]. Despite a high correlation between *C. sinensis* infection and hepatobiliary diseases established by both experimental and epidemiological evidence, the literature provides limited data that can be used to elucidate the molecular mechanism underlying the parasitic biology and pathogenesis of *C. sinensis* infection.

Chronic hepatobiliary damage is thought to result from chronic mechanical irritation of the epithelium by fluke suckers, metabolites, excretory-secretory products (ESP) and tegumental molecules [3]. These studies encouraged further investigations into drug design and vaccine trials to combat *C. sinensis* infection. Functional genomic studies could provide detailed information to explain the remaining questions regarding parasitic biology of *C. sinensis*, such as tissue tropism, energy sources and pathogenesis. In recent years, transcriptome data sets for *C. sinensis* generated by next-generation sequencing technology have provided a better understanding for the mechanism of this carcinogenic parasite [2]. In this study, to further elucidate the mechanism underlying the parasitism and pathogenesis of *C. sinensis* infection, we present the genome sequence of *C. sinensis* based on our previously generated draft genome [12]. In addition, the transcriptomes of multiple individual tissues (sucker, muscle, ovary and testis) of *C. sinensis* were sequenced to identify the molecular mechanism of parasitic features. The genome and transcriptome data generated herein could help to develop a more comprehensive understanding of the carcinogenic liver fluke and, more importantly, aid the research community in developing improved tools for the treatment and eradication of this neglected tropical disease.

## Materials and Methods

### Ethics Statement

*C. sinensis* flukes were isolated from naturally infected cats (Guangdong Province, China) for sample preparation. All experimental procedures were approved by the Animal Care and Use Committee of Sun Yat-sen University (Permit Numbers: SCXK (Guangdong) 2009–0011).

### New Assembly and Reannotation of the Genome

In addition to the paired-end data published previously [12], we sequenced two paired-end libraries of genomic DNA extracted from the same individual used in our previous study about draft genome and two mate-pair libraries of pooled genomic DNA extracted from twenty adult worms of *C. sinensis*. The scaffolds were constructed using a step-by-step method. Protein-coding genes were predicted using a combination of similarity-based, *ab initio* methods and genome-guided RNA-Seq assemblies. Repetitive element annotation, coding predictions and gene model annotation were performed as per the methods in our previous paper. The details and any associated references are available in Note S1.

### Construction of RNA Sequencing Libraries

Twenty adult *C. sinensis* flukes were isolated from cat livers (Guangdong Province, China) and rinsed several times with PBS. To prepare tissue-specific RNA, 2000 living adult flukes were split into four plates. Individual tissues, oral sucker, muscle, ovary and testis, were carefully dissected from the adult flukes with precision under a dissecting microscope with a fine micro-dissecting needle, separated and collected. The individual tissues were pooled separately, and total RNA was extracted using Trizol (Invitrogen, Carlsbad, CA, USA) in accordance with the manufacturer’s instructions.

The sequencing library was constructed as described in the TruSeq RNA SamplePrep Guide (Illumina). The fragments were enriched by PCR for 15 cycles and excised at 400 bp on a 2% agarose gel as the final libraries. A paired-end sequencing run was then performed on the Genome Analyzer IIx (Illumina, San Diego, CA, USA) in accordance with the Genome Analyzer IIx User Guide (Illumina). After masking adaptor sequences and removing contaminated reads, a total of 21.65 Gb of cleaned paired-end reads were produced.

### RNA-Seq Data Analysis

RNA-Seq data from four tissues (sucker, muscle, ovary and testis) were filtered by Fastx-tools [13] using the following criteria: 1) reads containing sequencing adaptors were removed; 2) nucleotides with a quality score lower than 20 were trimmed from the end of the sequence; 3) reads shorter than 50 bp were discarded; and 4) artificial reads were removed.

For each tissue, clean RNA-Seq data were assembled using Velvet [14], and contigs were clustered using TGICL [15]. The ESTs used in our previous study [12] and one by Korean researchers [2] were clustered using TGICL, and 454 sequencing data [3] were assembled by the Newbler Assembler. All of the above assemblies were clustered using CAP3 [16], and putative, full-length CDS for gene annotation were predicted using OrfPredictor [17].

RNA-seq reads were mapped to the genome using TopHat [18], and expression level of gene represented by value of fragments per kilobase of exon model per million (FPKM) was estimated using Cuffdiff [19], [20] according to our gene models using default parameters. An FPKM filtering of 1.0 in at least one of the four tissues was used to determine expressed genes. Genes that were determined to be expressed significantly differently in at least one of the four comparisons (testis *vs.* ovary, testis *vs.* muscle, ovary *vs.* muscle, and sucker *vs.* muscle) were considered to be differently expressing genes (DEGs). The DEGs were normalized by total expression in the four tissues and then clustered by the gplots package in R, followed by Gene Ontology (GO) enrichment analysis performed by BinGO [21] with Hypergeometric test and Benjamini & Hochberg False Discovery Rate (FDR) correction at a significance level of 0.01.

Gene structures, alternative splicing events and transcriptome profiles were visualized by using Gbrowse software [22]. Users can log in to the website of http://fluke.sysu.edu.cn/CsinGD/home.php, which allows users to navigate by scaffold coordinates, gene or transcript IDs.

To understand how adult liver flukes obtain enough energy, KEGG reference pathways were used to analyze the energy metabolism gene network by comparing the genome and transcriptome of *C. sinensis* with the genomes of *S. japonicum* and *S. mansoni*.

### Real-time Quantitative-PCR (qPCR) Validation of the DEGs

In addition, we used qPCR to selectively validate five genes that were differentially expressed between the oral sucker and the rest of the worm body. Total RNA was extracted from the oral sucker and from the rest of *C. sinensis* body using the Trizol RNA isolation protocol (Invitrogen, Carlsbad, CA, USA). First-strand cDNA synthesis was performed using Reverse-Transcriptase Superscript (TaKaRa Bio Inc., Japan) with the same quantity of total RNA as the template. The primers employed for amplification of the five genes and β-actin (gene for internal control) were shown in Table 1. Real-time PCR amplification was performed using a LightCycler480 (Roche, Switzerland) and a SYBR Premix ExTaq Kit (TaKaRa Bio Inc., Japan). The PCR amplification program was 95°C for 30 sec, followed by 40 cycles of 95°C for 5 sec and 60°C for 20 sec. After amplification, a melting curve was performed using a program of 95°C for 30 sec and 65°C for 15 sec, followed by an increase to 95°C while continuously collecting the fluorescence signal. The LightCycler480 software (version 1.5) was used to analyze the data according to the 2^−ΔΔCt^ method [23]. The amplification of DNA from the whole body, excluding the oral sucker, was employed as the calibrator to evaluate relative expression levels.

**Table 1:** Primers for qRT-PCR.

Genes	Forward primer	Reverse primer
β actin	5′ ACCGTGAGAAGATGACGCAGA 3′	5′ GCCAAGTCCAAACGAAGAATT 3′
csin103480	5′ CTGGGAGGATGGAGTTTG 3′	5′ GCTGCCGTTGTATTTCAC 3′
csin103125	5′ TCCGTGCGACAGTATTCC 3′	5′ CTTGTCCACCACCTTTGC 3′
csin103126	5′ TTTGTTGTGGCAGTGGGT 3′	5′ CTGGAACTTTGCCGATGA 3′
csin112707	5′ CGGGCAGGAAGGAACTA 3′	5′ TCCACGCAGACGAATGT 3′
csin102672	5′ CGAAGACGGGAAAGGTGA 3′	5′ GGACATTGTGGCGTGAGA 3′

### Prediction of Excretory-secretory Proteins (ESPs)

ESPs were chosen by three criteria [24]: 1) the probability of signal peptides and signal anchors as predicted by SignalP 3.0 [25]; 2) a lack of transmembrane helices as predicted by TMHMM 2.0 [26]; and 3) identification as a secretory pathway signal peptide (SP) as predicted by TargetP 1.1 [27]. Gene Ontology was used to annotate the functions of putative ESP genes.

### Positive Selection

*Opisthorchis viverrini* and *Clonorchis sinensis* were chosen for positive selection detection. For *O. viverrini*, transcripts were assembled using 454 Newbler software. Pairwise alignments between the two species were extracted by BLAST using bi-directional best hits, followed by ClustalW re-aligning. Phylogenetic Analysis Using Maximum Likelihood (PAML) was performed to calculate selection pressure using a sliding window method. The window size was 60 nucleotides, and the step size was 9 nucleotides.

### Data Accessibility

This whole-genome shotgun project was also deposited at DDBJ/EMBL/GeneBank under the project accession [BADR00000000]. The version described in this article is the updated version, BADR02000000 with Contigs [BADR02000001-BADR02006190] and Scaffolds [DF142828-DF145382]. The genomic sequencing data were deposited in the NCBI Sequence Read Archive (SRA) under the project accession number [SRA029284]. The RNA-Seq data were deposited in the EBI ArrayExpress database under the accession number [E-MTAB-827]. All *C. sinensis* genome and transcriptome data have been released and can be accessed at the website: http://fluke.sysu.edu.cn/CsinGD/.

## Results and Discussion

### Genomic Features of *C. sinensis*

In the present study, two paired-end and two mate-pair libraries of *C. sinensis* adult were sequenced. In total, 263.38 million raw pairs of sequence reads (107X coverage) were produced, with 221.62 million pairs (63X coverage) used in the genome assembly after data filtering (Table S1). The update version was re-assembled with all reads. Due to the higher depth of short insert-size paired-end sequencing data and the additional long insert-size mate-pair sequencing data, genes in the update version had better continuity than those in the previous version (Table 2). The scaffolds number decreased from 26,446 to 4,348, and the scaffold N50 size had a great improvement which was from 43 kb to 417 kb, almost ten-fold longer than that of previous version. BLAST tools were employed to generate the alignment of these two genome versions. It happened frequently that larger size scaffolds in update version actually covered several scaffolds of previous sequencing data. A total of 13,634 gene models, of which 79.6% could be annotated by six databases (Swiss-Prot, KEGG, COG, InterPro, GO and NR), were identified as the final gene set (Table 3, Table S2). The average length of gene increased from 11,548 bp in previous version to now 17,797 bp owning to larger scaffold N50 size. Additionally, the average number of exons in genes was 6.9 in the update genome, while that was 5.9 in the previous version, which indicated that structural integrity of genes in new version was superior to the last one. It might due to that shorter scaffold/contig N50 size would lead to split of gene during prediction of gene model so that one gene would be splitted into two genes in old version. For example, the longest scaffold (scf00011) of the new version was composed of 119 short scaffolds from the last one. The increased percentage of genes with structural integrity resulted in more precise prediction of CDS and gene model in the update version. The GC content was calculated to be approximately 43% (Figure S1). In *C. sinensis*, approximately 32% of the genome represents interspersed repeats, based on both known and *ab initio* repeat libraries (Figure S2). Details of the assembly and annotation of the upgraded genome are shown in Note S1.

**Table 2:** Summary of the *C. sinensis* genome assembly.

Genome version	Contig & Scaffold	Total length (Mb)	Contig & Scaffold no.	N50* (bp)	N90* (bp)	Longest (bp)
Ver. 2	Contig	547.10	6,190	233,037	61,404	1,089,335
	Scaffold	547.29	4,348	417,486	101,303	2,050,842
Ver. 1	Contig	515.56	60,796	14,708	4,079	137,874
	Scaffold	516.47	26,446	42,632	8,441	400,764

Ver. 2 and Ver. 1 indicate the upgraded genome and the previously published genome, respectively.

* N50 and N90 size of contigs or scaffolds were calculated by ordering all sequences and then adding the lengths from the longest to the shortest until the summed length exceeded 50% and 90% of the total length of all sequences.

**Table 3:** General pattern of *C. sinensis* protein-coding genes compared with *S. mansoni* and *S. japonicum.*

Species	Gene model (#)	Average gene length (bp)	Average protein length (bp)	Average exon length (bp)	Average exon (#)	Average intron length (bp)	CDS proportion (%)	Intron proportion (%)
*C. sinensis* Ver. 2	13,634	17,797	531	232	6.9	2,761	3.96	40.3
*C. sinensis* Ver. 1	16,258	11,548	441	223	5.9	2,077	4.14	32.2
*S. japonicum*	12,657	9,999	392	222	5.3	2,059	3.70	28.00
*S. mansoni*	11,747	13,395	446	222	6	2,407	4.10	37.20

### Tissue-specific Transcriptome Analysis

To discover new transcribed isoforms and to comprehensively characterize their expression dynamics among different tissues, RNA-Seq was performed on the oral sucker, muscle, ovary and testis tissues of *C. sinensis*. In total, approximately 117.32 million pairs of 121-bp paired-end reads were produced using the Illumina Genome Analyzer IIx platform. After filtering, 98.69 million read pairs were retained for further analysis (Table S3). Based on the 13,634 identified gene models, 27,082 transcript isoforms (1.98 isoforms per gene) were predicted by using RNA-seq data. Approximately 26% (3,535/13,634) of the gene models had two or more transcribed isoforms (Table S4). In total, 14,087 alternative-splicing events grouped into 11 different splicing patterns were detected (Table S5). In addition, 4,259 transcribed regions corresponding to 4,821 transcripts were newly identified.

**Figure 1:**
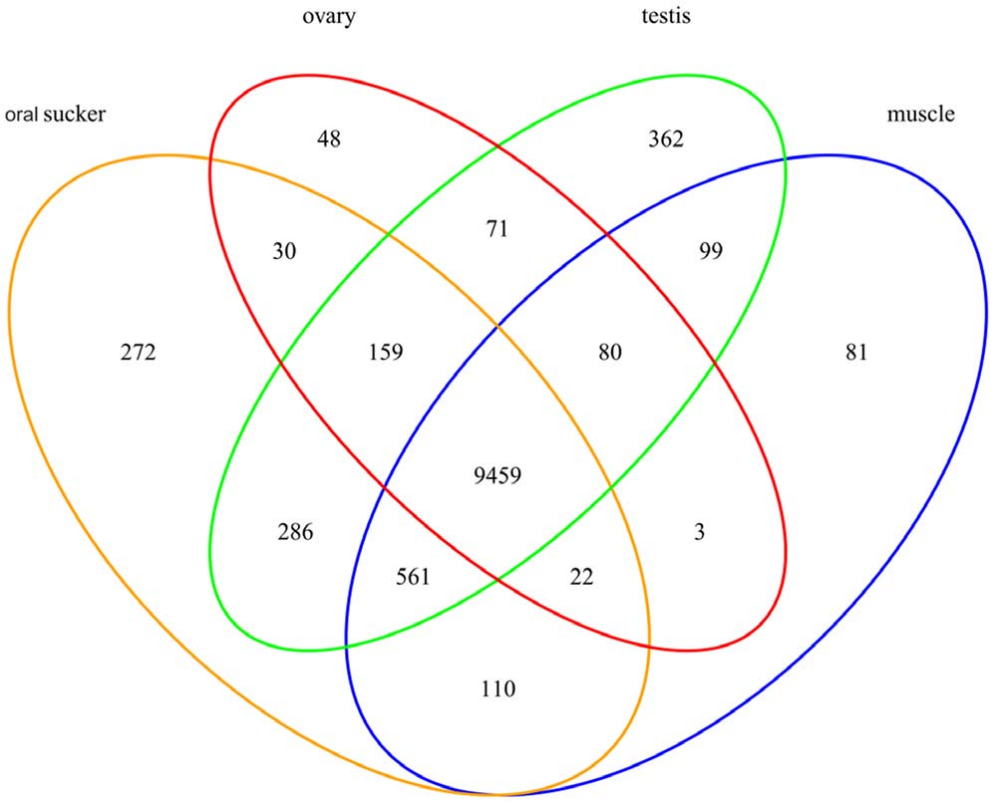
Transcriptome analysis of genes expressed specifically in different tissues. Footnote: Genes with fragments per kilobase of exon per million fragments mapped (FPKM) >1 were considered to be expressed**.** Orange ovals, red ovals, green ovals and blue ovals indicated the genes expressed in oral sucker, ovary, testis and muscle, respectively. There were 272, 51, 648, and 81 genes expressed only in the oral sucker, ovary, testis and muscle, respectively. There were 9459 genes expressed in all four tissues.

**Figure 2:**
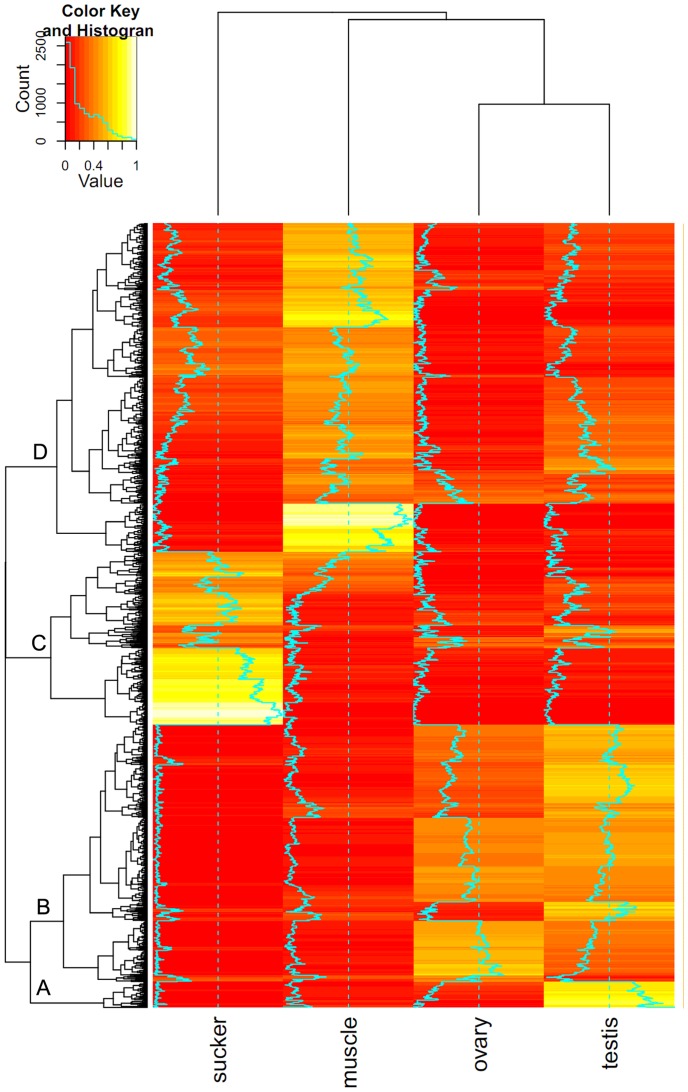
Heat map of differentially expressed genes (DEGs) in the four tissues. Footnote: The DEGs were divided into four classes. Class A represented genes with high expression in testis Class B represented genes highly expressed in both the testis and ovary, Class C represented genes highly expressed in the in oral sucker and Class D represented genes highly expressed in muscle.

The genes expressed specifically in each tissue were identified (Figure 1), in total, the expression of 9,860 genes and 19,435 transcripts with more than one FPKM were detected in at least one tissue (Table S6). The majority of genes were expressed with an FPKM ranging from 1 to 200 in the four tissues (Table S7; Figure S3). Of 13,634 gene models, 9459 genes (69.4%) expressed in all four tissues from the adult worm. The result suggested the tissue-specific and life stage-specific expression of *C. sinensis* genes. The Cuffdiff program was used to identify DEGs: 1,094 in muscle vs. oral sucker, 1,315 in muscle vs. ovary, 1,043 in muscle vs. testis and 516 in ovary vs. testis. Of those DEGs, 2,583 were selected and clustered into four classes (Figure 2). Some GO terms enriched by DEGs had a statistically significant over-representation (corrected *p* value <0.01) (Table S8).

**Figure 3:**
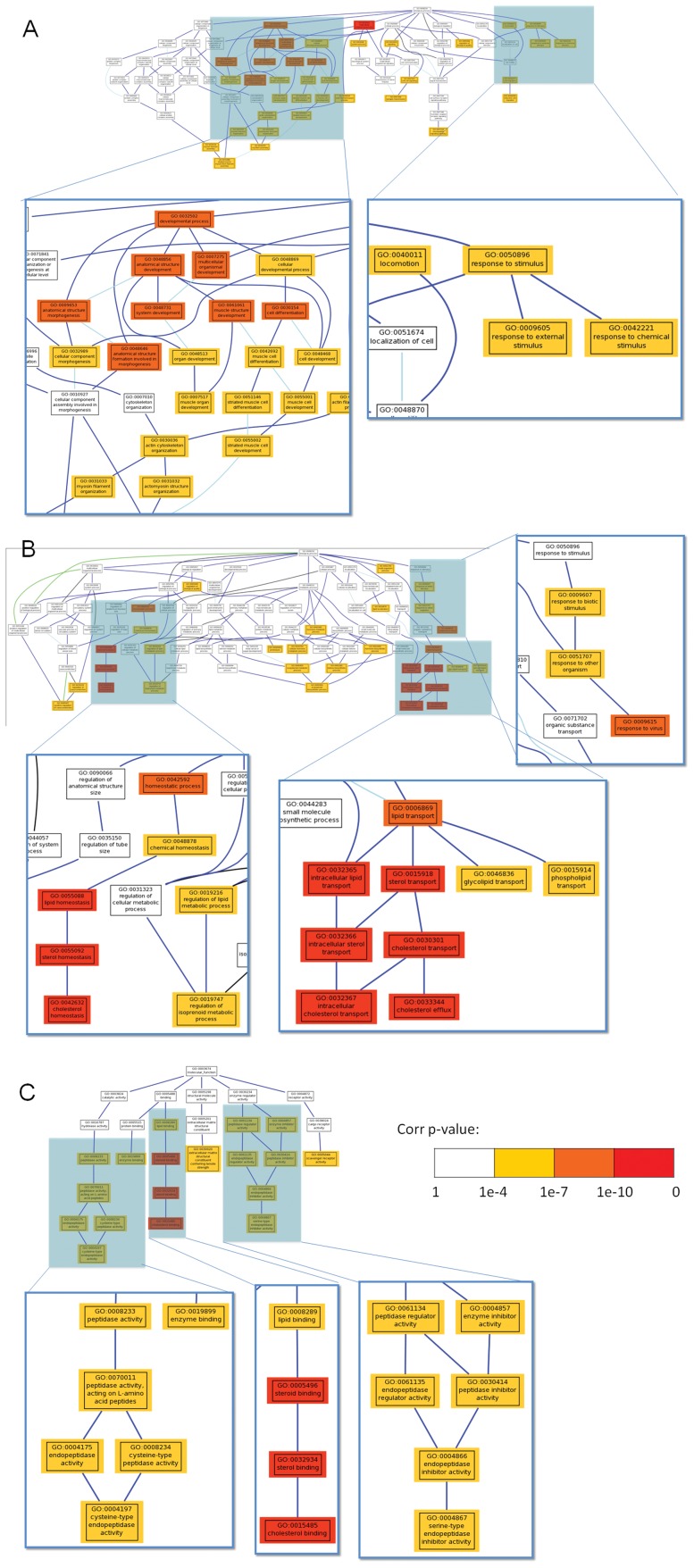
The functional enrichment of highly expressed genes in the oral sucker and genes encoding excretory-secretory proteins (ESPs). Footnote: (A) The highly expressed genes in the oral sucker were involved in two categories: response to stimulus and muscle-related development. The level of significance is indicated by different colors. (B) For the GO biological process category, genes encoding ESPs were enriched in the pathways of response to biotic stimulus, lipid transport and lipid homeostasis. (C) For the GO molecular function category, genes encoding ESPs were enriched in the pathways of peptidase activity, enzyme inhibitor activity and lipid binding.

Genes with high expression in testis (Class A, Figure 2) were enriched in microtubule-based movement, microtubule-based processes, negative regulation of actin filament polymerization and negative regulation of protein polymerization categories, while genes highly expressed in both testis and ovary (Class B, Figure 2) were enriched in spermatogenesis, sperm motility, male gamete generation and fertilization pathways. Six tektin genes were highly expressed in both testis and ovary. The tektin family includes Tektin-1 (csin106473, csin103551), Tektin-2 (csin102705), Tektin-3 (csin111271, csin112518) and Tektin-4 (csin108281). Previous investigations have showed that tektins are involved in sperm motility and human male infertility [28]. Taken together, these results suggest that many genes in Classes A and B could have sex-related functions. Genes highly expressed in the oral sucker (Class C, Figure 2) were enriched in pathways related to lipid binding, stimulus response and muscle differentiation (Figure 3A), while genes highly expressed in muscle (Class D, Figure 2) were enriched in pathways related to metabolic function.

### Abundantly Expressed Genes in the Oral Sucker and *C. sinensis* Migration

Unlike the muscle, ovary and testis transcriptomes, the most abundantly expressed genes in the oral sucker (Class C in Figure 2) transcriptome were related to response to stimuli and muscle development (Figure 3A; Table S8).

**Figure 4:**
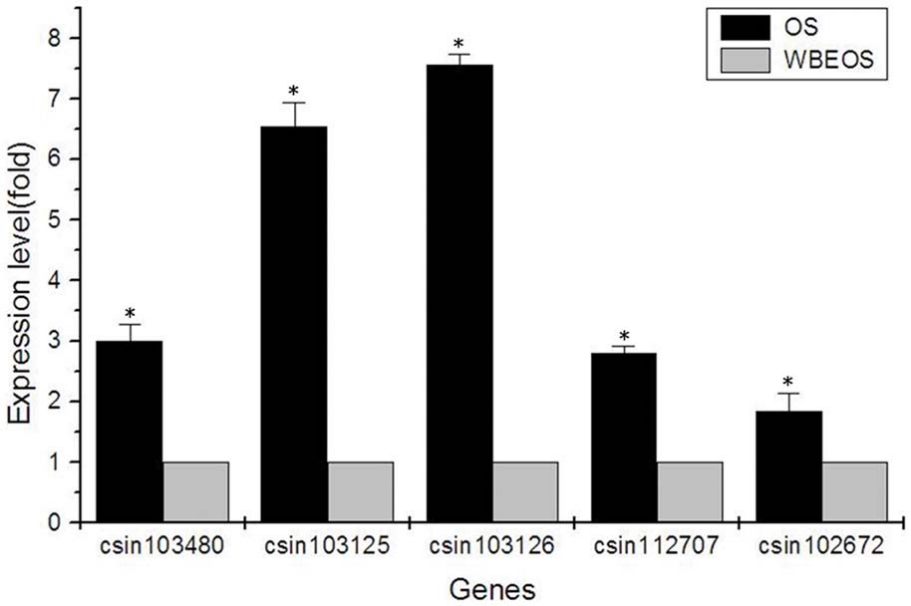
qPCR analysis of genes encoding proteins with an MD-2-related lipid-recognition domain. Footnote: The expression levels of five genes containing MD-2-related lipid-recognition domains (csin103480, csin103125, csin103126, csin112707 and csin102672) in the oral sucker (OS) were significantly higher than that in the rest of the *C. sinensis* body (WBEOS) (P<0.05). The relative expression level of each gene was calculated as 2^−ΔΔCt^ normalized by the expression level of β-actin.

Some predicted DEGs in Class C which are assigned to the enriched GO category of response to stimuli such as csin112707, csin103125, csin103480, csin102672 and csin103126 were confirmed to statistically high expressed in oral sucker in the current study by qPCR (Figure 4). The selected genes encoding proteins which contain MD-2-related lipid-recognition domain are categorized to enriched GO subclass of chemical homeostasis, homeostatic process and biological regulation. The domain is mostly present in Niemann-Pick C2 protein (NPC2), also known as epididymal secretory protein E1 in human [29]. NPC2 protein is a small lysosomal glycoprotein able to bind to a range of cholesterol-related molecules with micromolar affinity [30]. In humans, NPC2 protein facilitated cholesterol recognition and transport [31]. 8 of 37 genes (Table S9) encoding NPC2 homologues moderately expressed (FPKM>20 in either tissue). Among 8 genes, the five genes mentioned above simultaneously are excretory-secretory proteins (Table S10). Considering that lipids including cholesterol and phospholipids are major components of bile, NPC2 homologues might be play roles in chemical homeostasis of *C. sinensis* and facilitate the adult to parasite in bile tract [32]. Together with that they highly express in the oral sucker, it is possible that the oral sucker may participate in the response to chemical stimuli. It is worthy of further study.

GO annotation indicates that most abundantly expressed genes in the oral sucker are involved in muscular movement, involving pathways that affect the cytoskeleton, the muscle structure, calcium ion binding and motor activity (Table S8). We have confirmed that myophilin-like protein (csin111322) which is assigned to the GO subcategory expresses prominently in oral sucker rather than other tissues [33]. Ultrastructural observations of trematodes have shown that oral and ventral suckers consist of an extensive complex of circular and longitudinal muscles that facilitate attachment and migration along the tissue surfaces [34]. The high expression levels of motorial molecules in the oral sucker of *C. sinensis* provide strong evidence that trematode oral suckers are the key attachment organ during parasite invasion and migration.

In addition, previous investigations into trematode nervous systems have shown that suckers play an important role in directing parasite behaviors [35]. A large number of neuropeptides and nerve fibers predominantly exist within *C. sinensis* oral suckers, including neuroendocrine protein (csin108563), neuronal acetylcholine receptor (csin100235), and neurofilament (csin100217).

### Energy Metabolism-related Genes and *C. sinensis* Parasitism

#### The source of energy

**Figure 5:**
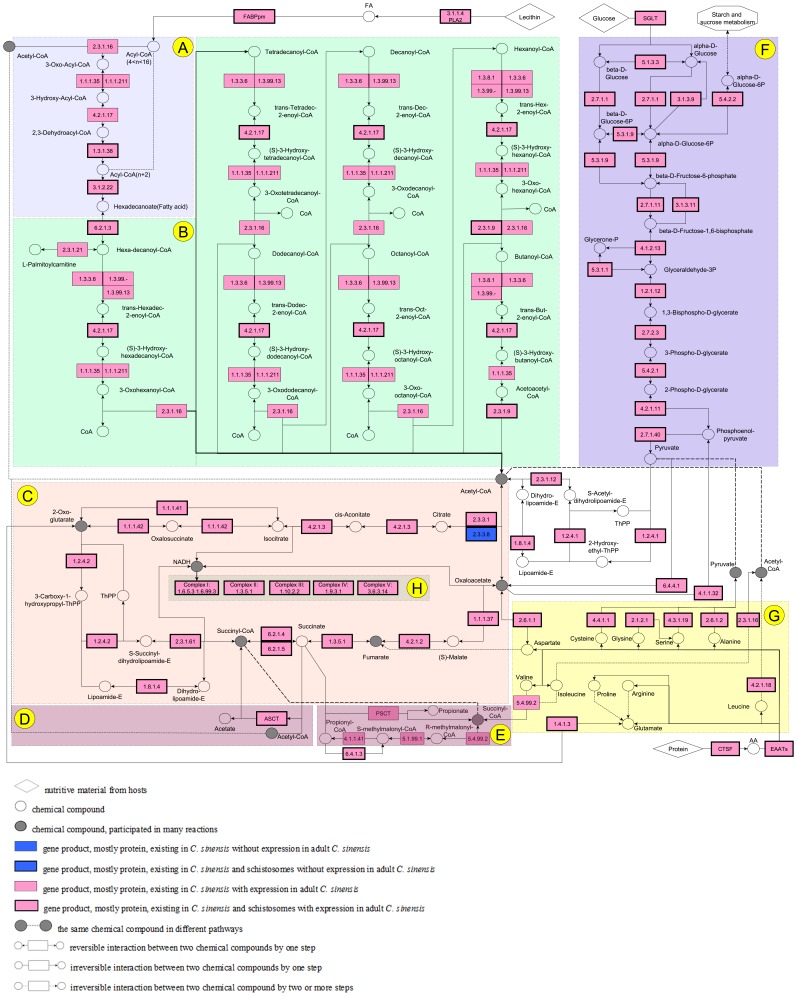
The *C. sinensis* metabolic pathway. The adult *C. sinensis* worm makes full use of bile fatty acids, sugars and amino acids as energy sources. All genes present in the citrate cycle and oxidative phosphorylation pathways were highly expressed, implying that the adult worm could obtain large amounts ATPs from aerobic respiration. (A) Fatty acid elongation. (B) Fatty acid metabolism. (C) Citrate cycle (TCA cycle). (D) Acetate:succinate cycle. (E) Fumarate reduction pathway. (F) Glycolysis/Gluconeogenesis. (G) Amino acid metabolism related to energy metabolism. (H) Oxidative phosphorylation.

As *C. sinensis* adults inhabit the intrahepatic bile duct, where there is a micro-aerobic and low-glucose environment, its energy sources and metabolic pathways are focal points of its parasitic biology. The energy metabolism gene network of *C. sinensis* was shown in Figure 5. The majority of genes involved in fatty acid elongation, fatty acid metabolism, the citrate cycle (TCA cycle), glycolysis, gluconeogenesis, oxidative phosphorylation and amino acid metabolism were expressed in *C. sinensis* (Figure S4; Table S11).

The energy requirements of many organisms are mostly satisfied with fatty acids. Trematode parasites, however, are thought to lack a *de novo* fatty acid synthesis pathway [12], [36], [37]. In parasites, FABPs are critical proteins that enable fatty acid transportation [38]. These proteins were highly expressed in *C. sinensis* and likely facilitate the ability of the adult liver fluke to efficiently utilize bile duct fatty acids. In our previous study, FABPs were characterized in the adult worm and were confirmed to localize in the vitelline gland, tegument, intestine and seminal vesicle [39].

Moreover, unlike *schistosoma*, KEGG pathway analysis showed that fatty acid elongation could occur within *C. sinensis*, as all enzymes involved in the pathway were expressed at high levels (FPKM>100) in adult worms. Additionally, the genes encoding the enzymes involved in the pathway that converts glucose to acetyl-CoA are expressed at high levels (FPKM>100) in each tissue. This observation is consistent with a previous study showing glucose serving as an energy source in *C. sinensis* [40]. Given these data, how does the adult worm obtain enough glucose in the bile duct? Seven genes encoding glucose transporters were identified. Among these genes, csin110076 was expressed in each tissue at high levels (FPKM>200), and csin110077 was expressed only in the sucker (FPKM = 337.5). These highly expressed glucose transporters might help the adult worm absorb blood glucose efficiently from capillaries destroyed in the bile duct epithelia when the adult invades the bile duct [41]. Meanwhile, all genes involved in gluconeogenesis were found in the *C. sinensis* genome (Figure 5F; Table S11). The gene (csin103490) encoding phosphoenolpyruvate carboxykinase (PEPCK, E4.1.1.32), which can transform oxaloacetate to phosphoenolpyruvate and play role in malic acid disproportionation [42], was expressed at high levels (FPKM>1,000) in each tissue, especially in the sucker and muscle (FPKM>2,000). In addition, fructose-1, 6-bisphosphatase (csin111764), a key regulatory enzyme of gluconeogenesis, highly expressed (FPKM>600) and was of efficient enzyme activity in *C. sinensis* adult (our unpublished data). Taken together, *C. sinensis* adult might have the ability of generating glucose from non-carbohydrate carbon substrates by gluconeogenesis.

Amino acids, such as cysteine, serine, alanine and glycine, that can be transformed to enter the TCA cycle, could also provide energy and metabolites (Figure 5G). The cathepsin F gene csin100047, a major component of the lysosomal proteolytic system [43], was expressed at extremely high levels in the adult worm (FPKM>15,000), indicating that *C. sinensis* could utilize proteins from the host.

#### Energy metabolic pathway

All genes encoding enzymes involved in the fatty acid β-oxidation pathway were found in the *C. sinensis* genome (Figure 5B), and these genes were expressed in multiple tissues of the adult liver fluke (Table S11). In contrast, this pathway is incomplete in *S. mansoni* and *S. japonicum*, with only four enzymes identified in these organisms. Three genes, K01692 (paaG), K07511 (ECHS1) and K07515 (enoyl-CoA hydratase), encoding enoyl-CoA hydratase (4.2.1.17), an enzyme that hydrates the double bond between the second and third carbons on acyl-CoA, were identified in *C. sinensis*. However, only one of these genes, K01692 (paaG), is present in the genomes of either *S. mansoni* or *S. japonicum*, which suggests that the main source of energy is different in these two types of parasites. Furthermore, all genes encoding enzymes involved in the TCA cycle were found in the *C. sinensis* genome (Figure 5C) and were expressed in multiple tissues at high levels (FPKM >200) (Table S11).

The above results suggest that adult *C. sinensis* can obtain energy from fatty acids via the β-oxidation/citrate cycle/oxidative phosphorylation pathway. This would result in a large amount of FADH2 and NADH, which could then be oxidized to produce ATP (Figure 5). In turn, this observation raises the question of how the adult *C. sinensis* obtains enough oxygen in the micro-aerobic bile duct environment. Three genes encoding globins, the reversible oxygen binders recognized as oxygen sensors involved in oxygen storage and transport [44], were found in the *C. sinensis* genome. Among these genes, myoglobin (csin111409) was expressed at high levels (FPKM>20,000) in all tissues, consistent with a previous study [45]. This gene has also been observed in other trematodes, such as *Isoparorchis hypselobagri*, *Paramphistomum epiclitum*, and *Gastrothylax* [44]. Globins from trematodes exhibit a higher oxygen affinity than those from nematodes and symbiotic plants [46]. Thus, the high level of globin expression in *C. sinensis* might enable the adult worm to recruit oxygen efficiently from its external environment and then utilize the oxygen in oxidative phosphorylation.

Under anaerobic conditions, previous studies have shown that *Fasciola hepatica*, another liver fluke, could utilize acetyl-CoA and succinate to generate succinyl-CoA and acetate [47]. This reaction is catalyzed by mitochondrial acetate:succinate CoA-transferase (ASCT) and is followed by ATP generation [40]. There were two putative ASCT homologies (csin101487 and csin101488) identified in the *C. sinensis* genome and expressed at high levels in each tissue (Table S12). ASCT was also suspected of having propionate:succinate CoA-transferase (PSCT) ability, enabling it to also utilize succinate and propionyl-CoA as substrates to generate propionate and succinyl-CoA [40] in the fumarate reduction pathway (Figure 5E). Therefore, these two anaerobic pathways might also provide energy for the adult *C. sinensis* when oxygen levels are insufficient for aerobic metabolism.

In summary, the genome and transcriptome data generated herein demonstrated that the *C. sinensis* adult worm can obtain energy from an array of pathways to adapt to the micro-aerobic and low-glucose environment of the bile duct. Compared with the other two parasites, *Schistosoma mansoni* in blood and *Ascaris suum* in intestine, the glycolysis, TCA cycle and oxidative phosphorylation pathways were similar, but the pattern of fatty acid-related gene expression was different (Figure S5).

### Secretory Genes and *C. sinensis* Pathogenesis

The excretory-secretory proteins (ESPs) of parasites have attracted attention in the research community because of their potential uses in the development of diagnostics, vaccines, and drug therapies [48]. In the *C. sinensis* genome, 297 genes encoding ESPs were found (Table S10). Based on enrichment analysis of the biological process and molecular function of the ESP genes, these genes were found to be enriched in lipid-binding and -transport, cysteine-type peptidase and peptidase inhibitor functions (Figure 3B and 3C). Approximately 11% (33/297) of ESP genes had hydrolase activity (Table S13). Nine of them, including cathepsin B, D and L, belonged to the cysteine protease family, and these have been shown to be ESPs in the *C. sinensis* adult by shotgun LC–MS/MS [49]. Six of these genes were expressed at high levels (FPKM>400), particularly csin103066 and csin3068 (FPKM>3,500). Cysteine protease family members play important roles in parasite invasion by degrading host proteins [50]. These putative ESPs provide potential targets for anti-parasitic drugs and/or vaccine candidates.

In addition, some ESPs might participate in the cell proliferation, response to stimuli or signal pathways. These types of genes have been shown to be related to immune system evasion as well as the triggering and development of cholangiocarcinoma resulting from adult worm inhabitation [8]. Although GO annotation classified nine ESPs as possibly being related to cell proliferation, only two molecules, granulin (csin103348) and prohibitin (csin113184), were expressed in the adult worm. Granulin was expressed at very high levels, especially in the sucker (FPKM = 26104.80). The granulin family has been shown to regulate cell growth in humans, including not only normal development and wound healing but also tumorigenesis [51]. *Opisthorchis viverrini*, another liver fluke, was reported to contribute to the development of bile duct cancer by secreting a granulin-like growth factor [52]. These data suggest that *C. sinensis* granulin might also take part in the development of cholangiocarcinoma. Secreted phospholipase A2 (sPLA2) which can transform phospholipids into lysophospholipids and fatty acids [53] is also a putative ESP of *C. sinensis*. Considering that phospholipids are a primary component of bile ducts and that *C. sinensis* can utilize fatty acids to generate energy, *C. sinensis* sPLA2 (*Cs*sPLA2) might participate in adult worm energy generation. Recent studies have also shown that the sPLA2 proteins of other parasites could be involved in tumorigenesis, inflammation, and invasion [54], [55]. In our previous study, we demonstrated that *Cs*sPLA2 played an important role in the initiation and development of hepatic fibrosis caused by *C. sinensis* [56]. Other studies have shown that sPLA2 could induce proliferation in astrocytomas [57]. Another group showed that sPLA2 promotes cell death at an early stage of chondrogenesis [58]. However, the function of *Cs*sPLA2 in physiology of *C. sinensis* and its pathogenesis in clonorchiasis are still unclear. Further investigations are needed to clarify its roles.

**Figure 6:**
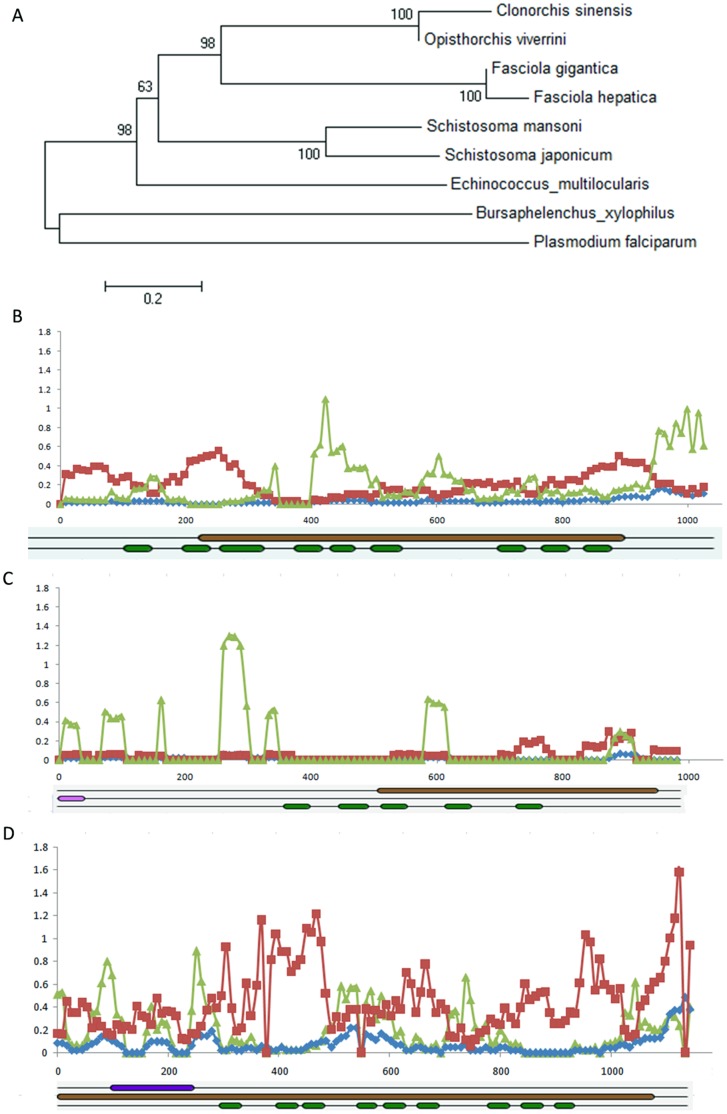
Evolutionary analysis of the signal peptide peptidase gene. Footnote: (A) Phylogenetic tree constructed from the SPP of different species including *C. sinensis* (csin112590), *O. viverrini* (Ov_Contig2040), *F. hepatica* (Contig19227), *Fasciola gigantica* (Fh_Contig1), *S. japonicum* (gi:256082245), *S. mansoni* (gi:226482537), *Echinococcus multilocularis* (gi:194212416), *Bursaphelenchus xylophilus* (BUX_s00862.17) and *Plasmodium falciparum* (PF3D7_1457000). Using a sliding window method, dN/dS was calculated in *C. sinensis vs. O. viverrini* (B), *Fasciola gigantica vs. Fasciola hepatica* (C), and *Equus caballus vs. Homo sapiens* (D). The window size was 60 nt and the step size was 9 nt. dN, dS and ω (dN/dS) were shown in blue, red and green lines, respectively. Bars in dark green represented transmembrane domains, and bars in brown represented peptidase A22B.

Signal peptides are required for protein secretion and are cleaved from presecretory proteins by signal peptidase and signal peptide peptidases (SPP) during or immediately after their insertion into the endoplasmic reticulum membrane [59]. It was reported that SPP interacts specifically and tightly with a large range of newly synthesized membrane proteins, including signal peptides, preproteins and misfolded membrane proteins [59]. SPP, an aspartic intramembrane protease that hydrolyzes signal peptide within the transmembrane domain of substrates, is implicated in several biological and pathological functions [60]. A phylogenetic tree was constructed using the SPPs of multiple different species including *C. sinensis*,*O. viverrini*, *F. hepatica*,*Fasciola gigantica*,*S. japonicum*,*S. mansoni*,and *Plasmodium falciparum* (Figure 6). Similarly, the SPP catalytic domain was also analyzed among these species, revealing that the intramembrane proteolysis of SPP is conserved in these species. The sequences of *C. sinensis* and *O. viverrini* shared the most similarity and were subjected to a dN/dS ratio sliding window calculation (the ratio of the number of non-synonymous substitutions per non-synonymous site to the number of synonymous substitutions per synonymous site). Regions between transmembrane domains were found to have a higher dN than dS, which indicated that these regions were acted upon by natural selection. This result was contrary to similar experiments performed in *Fasciola gigantica vs. Fasciola hepatica* and *Equus caballus vs. Homo sapiens*. It has been proposed that *Plasmodium falciparum* SPP could be a possible drug target in blood-stage malaria, as it is involved in merozoite invasion via host receptor binding [61]. The identification of SPP in the *C. sinensis* genome could indicate a method of pathogenesis based on secreted proteins. Further research into the possibility of inhibiting pathogenesis by blocking ESP release is warranted.

## Supporting Information

Figure S1 **GC content distribution in Clonorchis sinensis, Schistosoma japonicum, Schistosoma mansoni, Caenorhabditis elegans, and Schmidtea mediterranea.**
(DOC)

Figure S2 **The distribution of categories and the composition of repeat elements in the** ***C. sinensis*** **genome.** Footnote: SINEs: short interspersed elements; LINEs: long interspersed elements; LTR: long terminal repeat.
(DOC)

Figure S3 **The distribution of gene expression levels in four tissues.** Footnote: (A) muscle; (B) oral sucker; (C) ovary; (D) testis.
(DOC)

Figure S4 **Energy-related metabolism of** ***C. sinensis*** **.** Footnote: Both aerobic and anaerobic respiration pathways were observed in the adult fluke.
(DOC)

Figure S5 **Partial energy-related KEGG pathways of** ***C. sinensis*** **,** ***Schistosoma mansoni*** **and** ***Ascaris suum.*** Footnote: Green boxes indicated genes present in the genomes.
(DOC)

Table S1 **Main features of raw data from** ***C. sinensis*** **whole genome sequencing.** Footnote: We assumed that the genome size was 580 Mb.
(XLS)

Table S2 **Statistics for the reliable gene set with homology, functional annotation and putative full-length ORF support.**
(XLS)

Table S3 **Main features of the RNA-Seq data from four** ***C. sinensis*** **tissues.**
(XLS)

Table S4 **Distribution of numbers of transcribed isoforms for each gene model.**
(XLS)

Table S5 **Distribution of alternative splicing events in transcripts.**
(XLS)

Table S6 **Numbers of expressed genes and transcripts.**
(XLS)

Table S7 **Gene expression in four** ***C. sinensis*** **tissues.**
(XLS)

Table S8 **GO term enrichment of the DEGs classes.**
(XLS)

Table S9 **Transcription profile of genes containing MD-2 lipid recognition domains.**
(XLS)

Table S10 **Excretory-secretory products predicted by three tools.**
(XLS)

Table S11 **Expression of genes involved in energy-related pathways.**
(XLS)

Table S12 **Genome validation by transcriptome data.** Footnote: Assembled transcripts were anchored onto the genome as spliced alignments using BLAST.
(XLS)

Table S13 **Gene ontology of putative genes encoding excretory-secretory products.**
(XLS)

Table S14 **Non-coding RNA genes in the** ***C. sinensis*** **genome.**
(XLS)

Table S15 **Summary of** ***C. sinensis*** **miRNA precursors.**
(XLS)

Note S1 **Details of the upgraded genome.**
(DOC)
