# Supplementary material for: The Carcinogenic Liver Fluke, Clonorchis sinensis: New Assembly, Reannotation and Analysis of the Genome and Characterization of Tissue Transcriptomes
Source: PLoS One. 2013 Jan 30;8(1):e54732. doi: 10.1371/journal.pone.0054732 (PMC3559784; doi:10.1371/journal.pone.0054732)
Supplement: Figure S1 — GC content distribution in Clonorchis sinensis, Schistosoma japonicum, Schistosoma mansoni, Caenorhabditis elegans, and Schmidtea mediterranea. (DOC) [file pone.0054732.s001.doc]

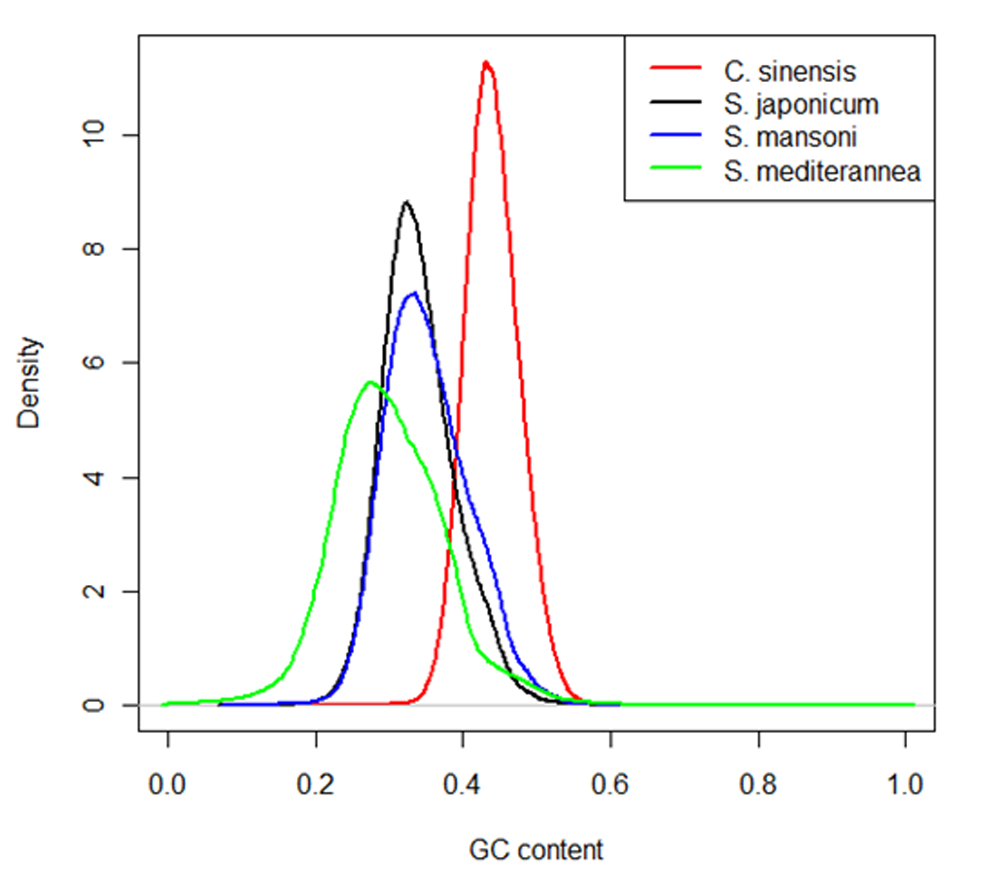


**Figure S1. GC content distribution of *Clonorchis sinensis, Schistosoma japonicum, Schistosoma mansoni, Caenorhabditis elegans,* and** ***Schmidtea mediterranea****.*
