# Supplementary material for: The Carcinogenic Liver Fluke, Clonorchis sinensis: New Assembly, Reannotation and Analysis of the Genome and Characterization of Tissue Transcriptomes
Source: PLoS One. 2013 Jan 30;8(1):e54732. doi: 10.1371/journal.pone.0054732 (PMC3559784; doi:10.1371/journal.pone.0054732)
Supplement: Figure S2 — The distribution of categories and the composition of repeat elements in the C. sinensis genome. Footnote: SINEs: short interspersed elements; LINEs: long interspersed elements; LTR: long terminal repeat. (DOC) [file pone.0054732.s002.doc]

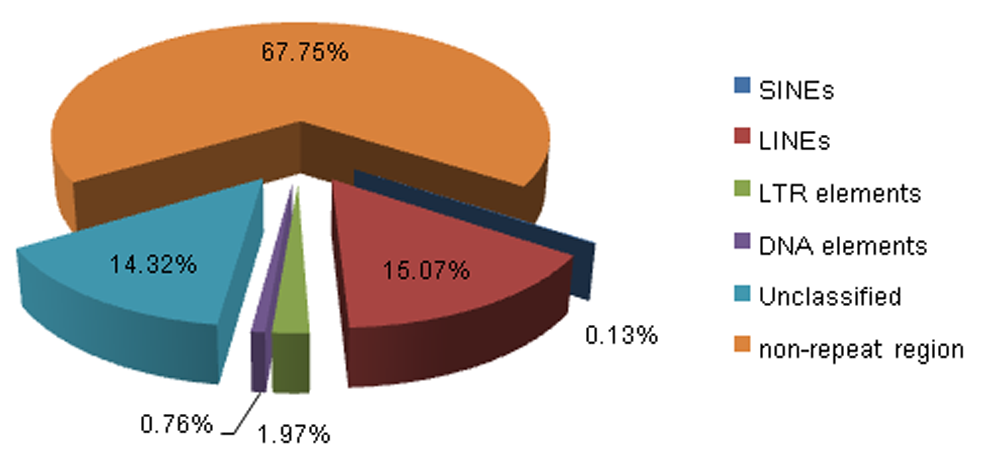


**Figure S2. The distribution of categories and composition of repeat elements in the *C. sinensis* genome.** SINEs: short interspersed elements; LINEs: long interspersed elements; LTR: long terminal repeat.
