# Supplementary material for: The Carcinogenic Liver Fluke, Clonorchis sinensis: New Assembly, Reannotation and Analysis of the Genome and Characterization of Tissue Transcriptomes
Source: PLoS One. 2013 Jan 30;8(1):e54732. doi: 10.1371/journal.pone.0054732 (PMC3559784; doi:10.1371/journal.pone.0054732)
Supplement: Figure S3 — The distribution of gene expression levels in four tissues. Footnote: (A) muscle; (B) oral sucker; (C) ovary; (D) testis. (DOC) [file pone.0054732.s003.doc]

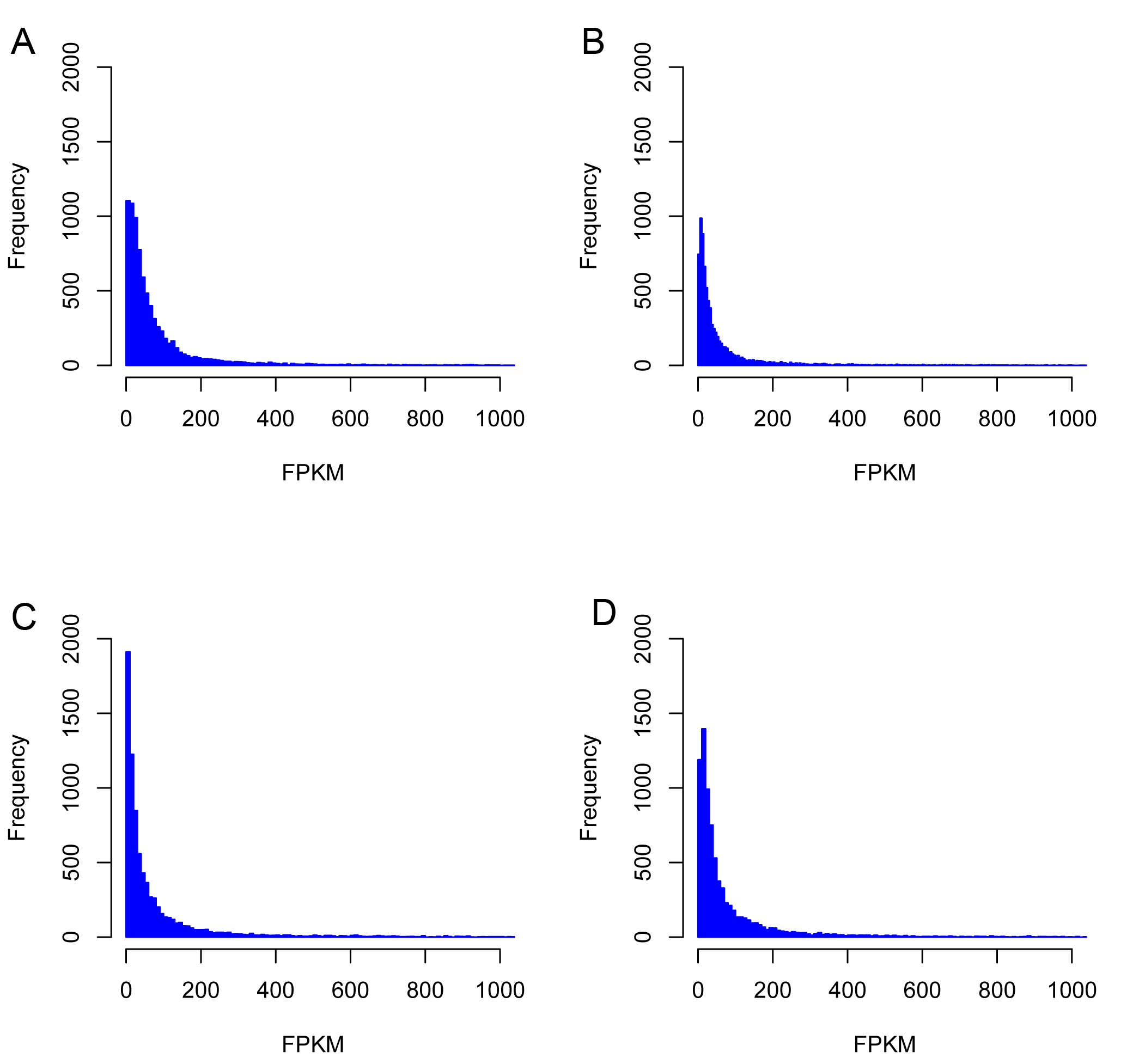


**Figure S3. The distribution of gene expression level in four tissues.** (A) muscle; (B) oral sucker; (C) ovary; (D) testis.
