# Supplementary material for: The Carcinogenic Liver Fluke, Clonorchis sinensis: New Assembly, Reannotation and Analysis of the Genome and Characterization of Tissue Transcriptomes
Source: PLoS One. 2013 Jan 30;8(1):e54732. doi: 10.1371/journal.pone.0054732 (PMC3559784; doi:10.1371/journal.pone.0054732)
Supplement: Figure S4 — Energy-related metabolism of C. sinensis . Footnote: Both aerobic and anaerobic respiration pathways were observed in the adult fluke. (DOC) [file pone.0054732.s004.doc]

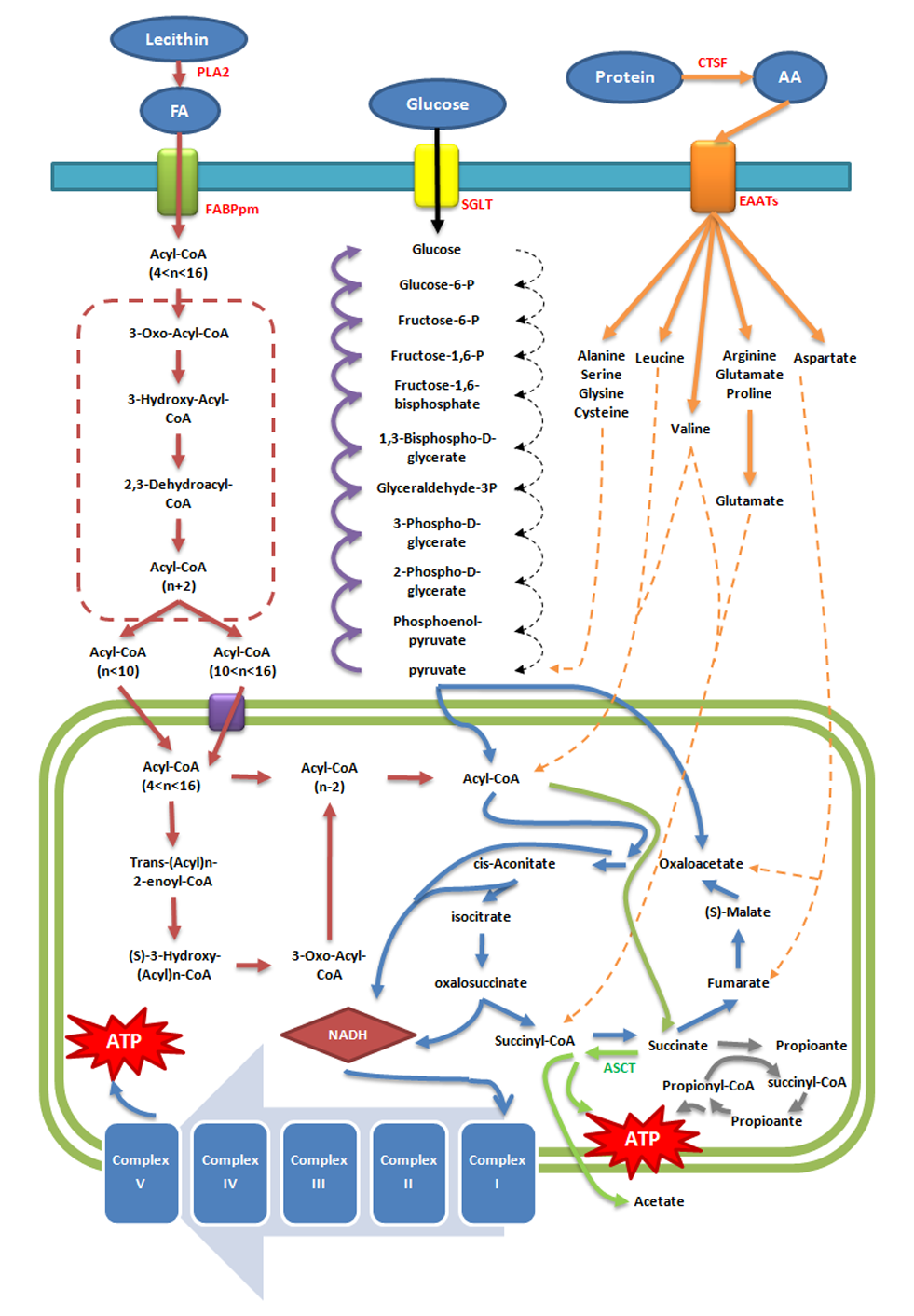


**Figure S4. Energy-related metabolism of *C. sinensis*.** Both aerobic and anaerobic respiration pathways were observed in adult fluke.
