# Supplementary material for: The Carcinogenic Liver Fluke, Clonorchis sinensis: New Assembly, Reannotation and Analysis of the Genome and Characterization of Tissue Transcriptomes
Source: PLoS One. 2013 Jan 30;8(1):e54732. doi: 10.1371/journal.pone.0054732 (PMC3559784; doi:10.1371/journal.pone.0054732)
Supplement: Figure S5 — Partial energy-related KEGG pathways of C. sinensis , Schistosoma mansoni and Ascaris suum. Footnote: Green boxes indicated genes present in the genomes. (DOC) [file pone.0054732.s005.doc]

|  | *Clonorchis sinensis* | *Schistosoma mansoni* | *Ascaris suum* |
| --- | --- | --- | --- |
| Glycolysis/ Gluconeogenesis | 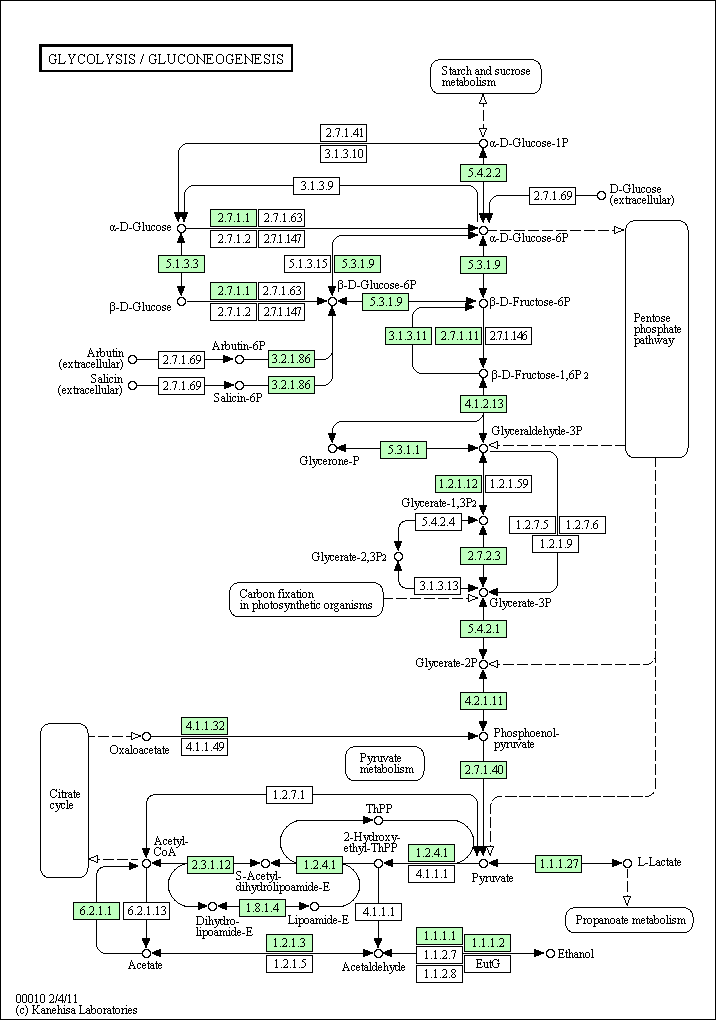 | 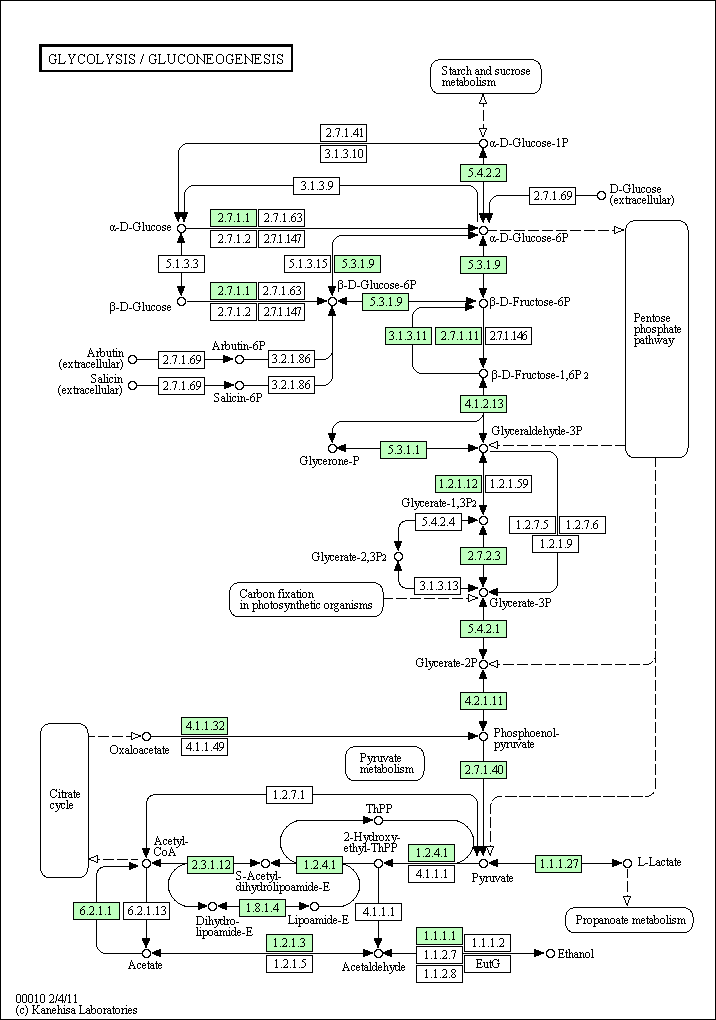 | *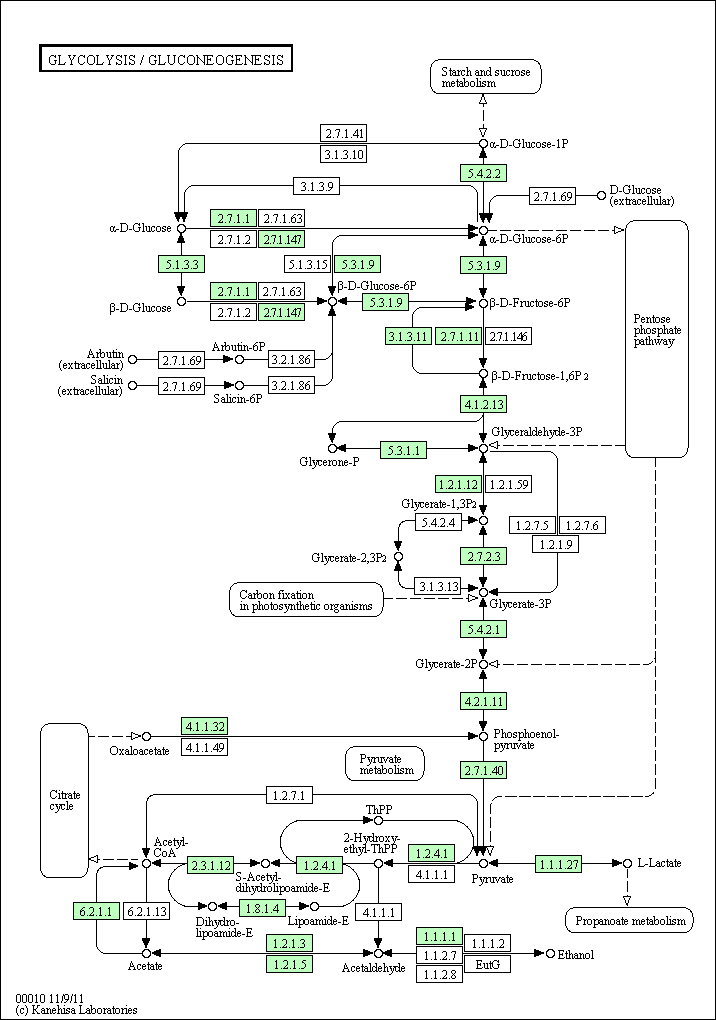* |
| TCA cycle | 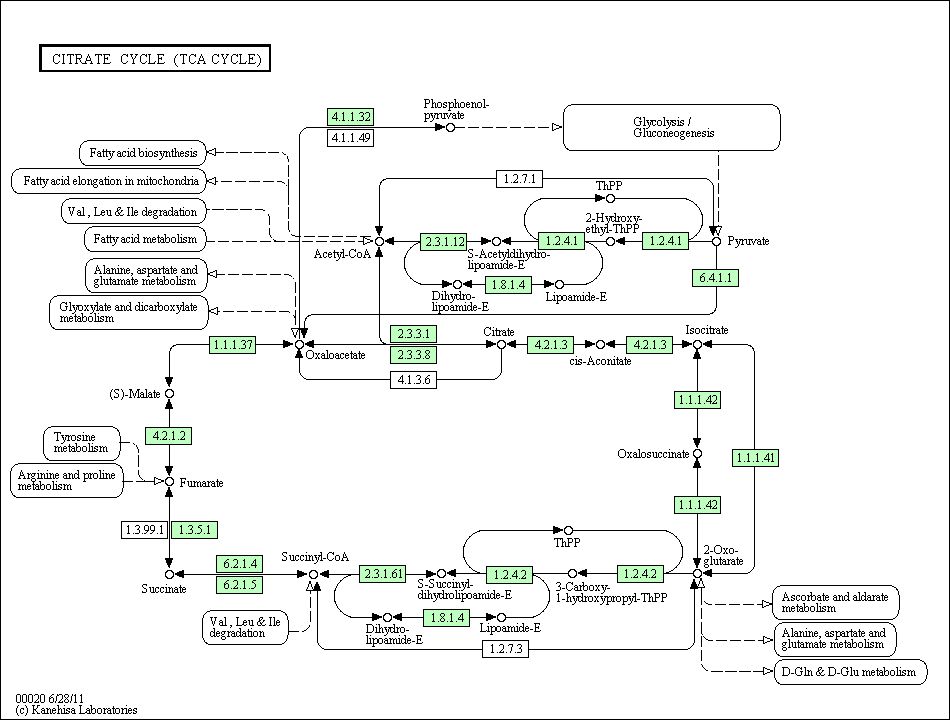 | 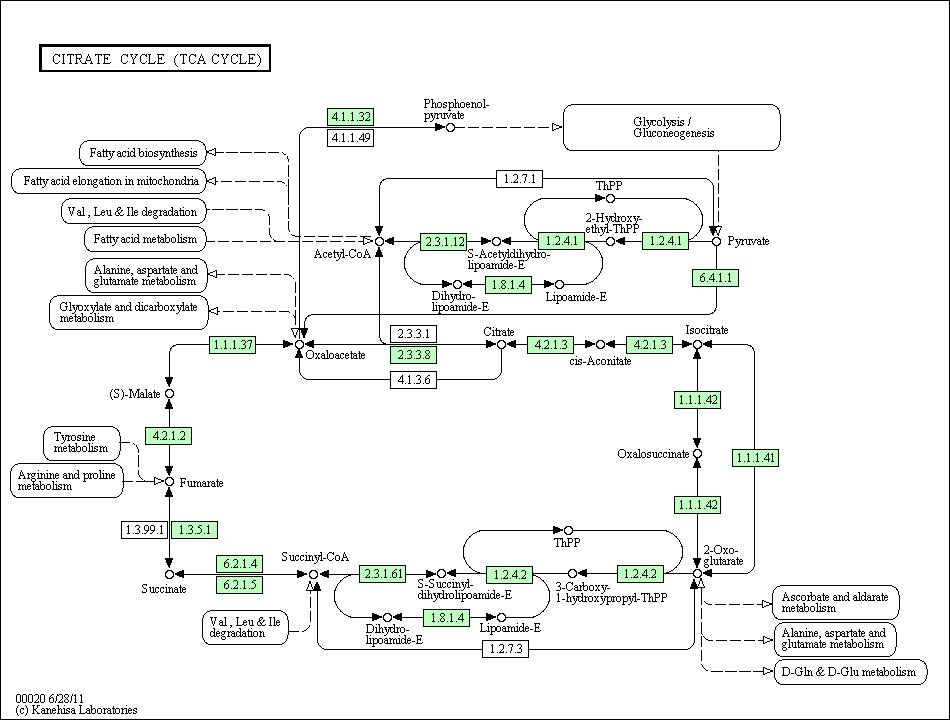 | 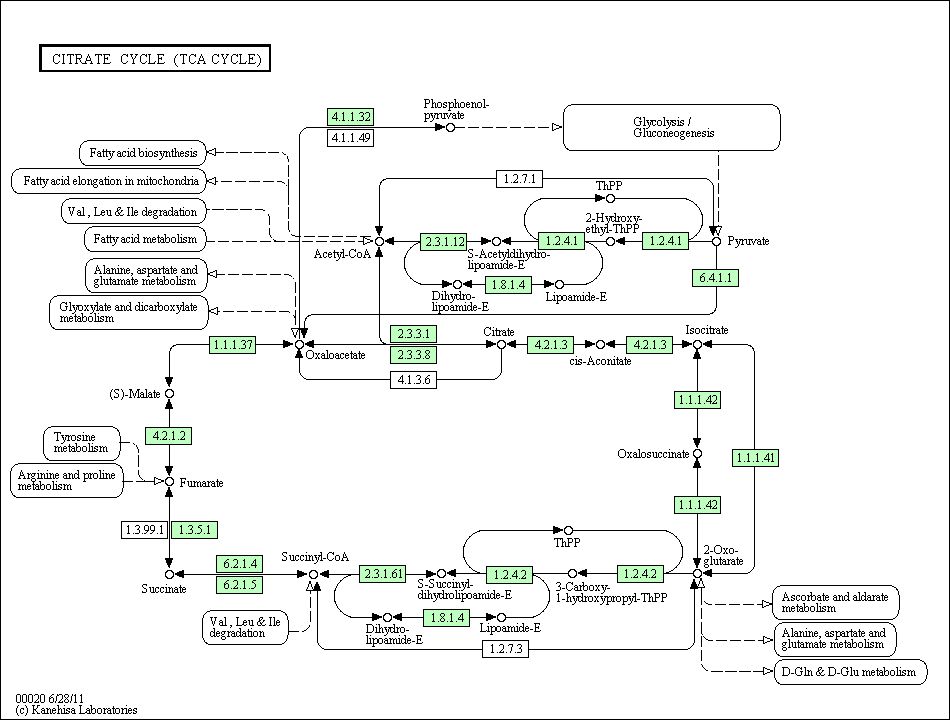 |
| Oxidative phosphorylation | 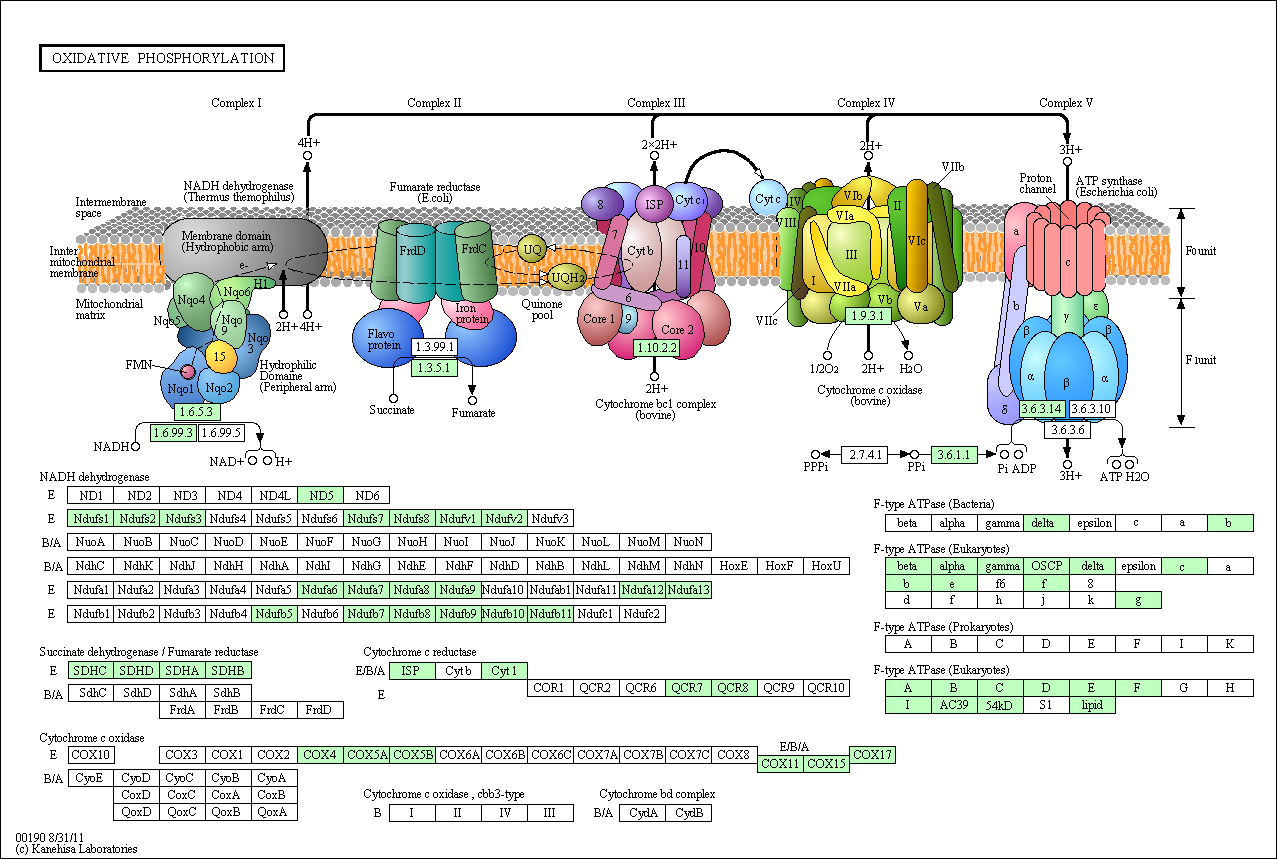 | 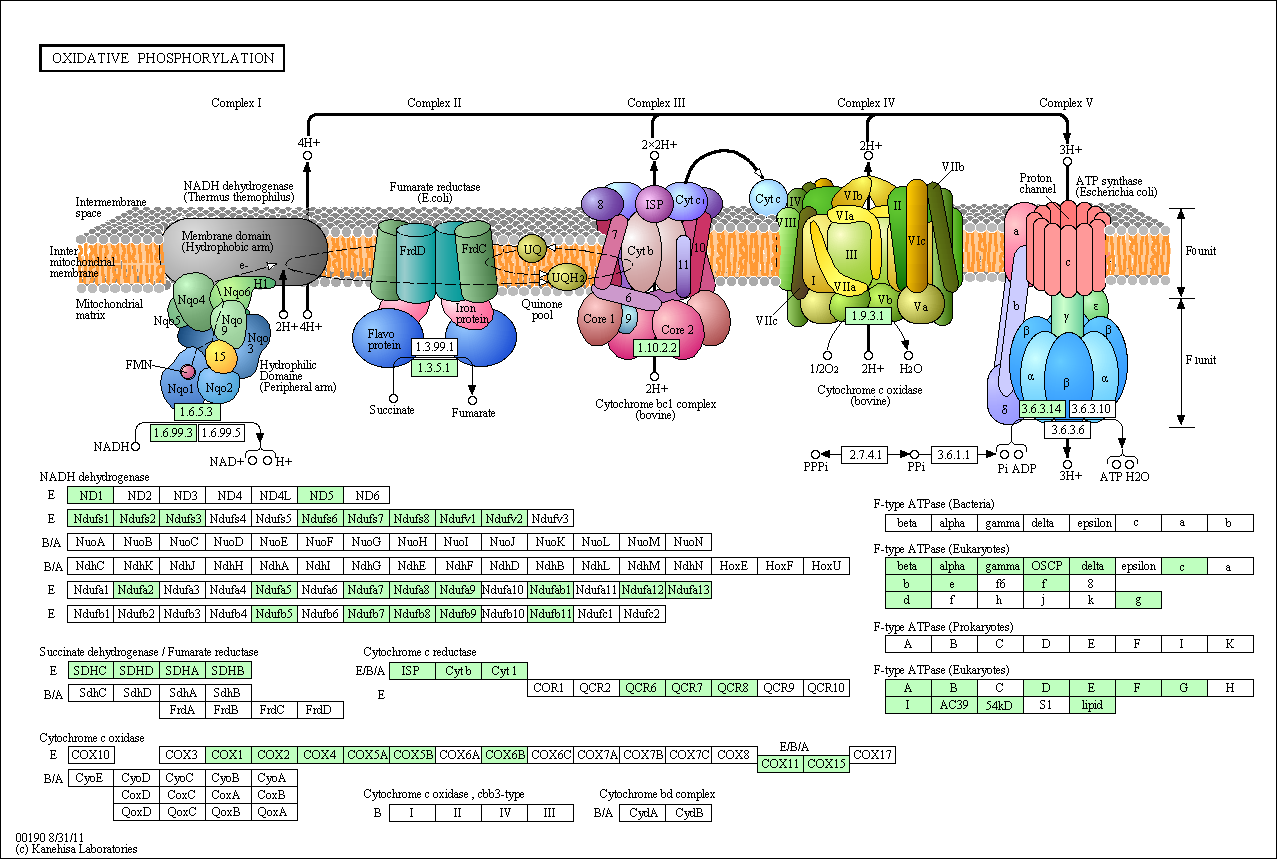 | 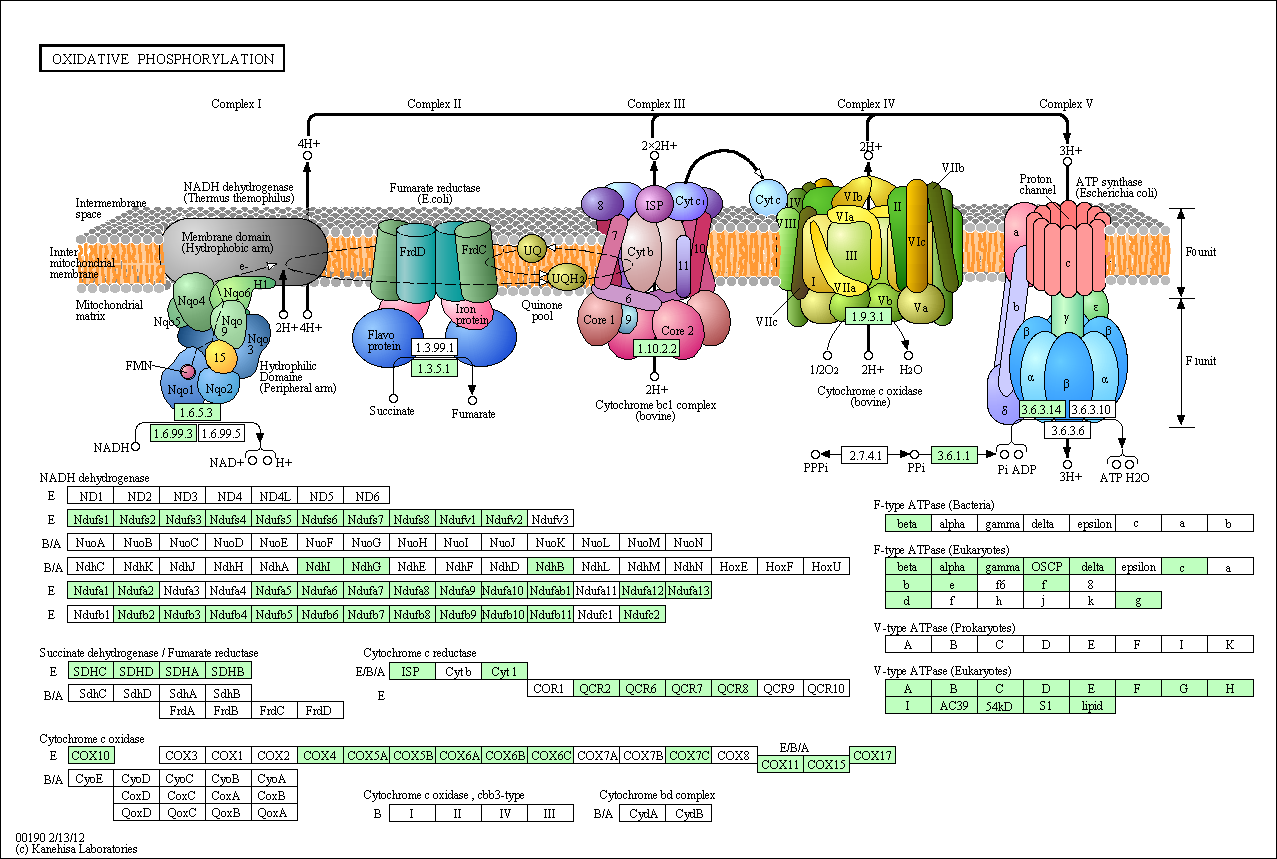 |
| Fatty acid elongation | 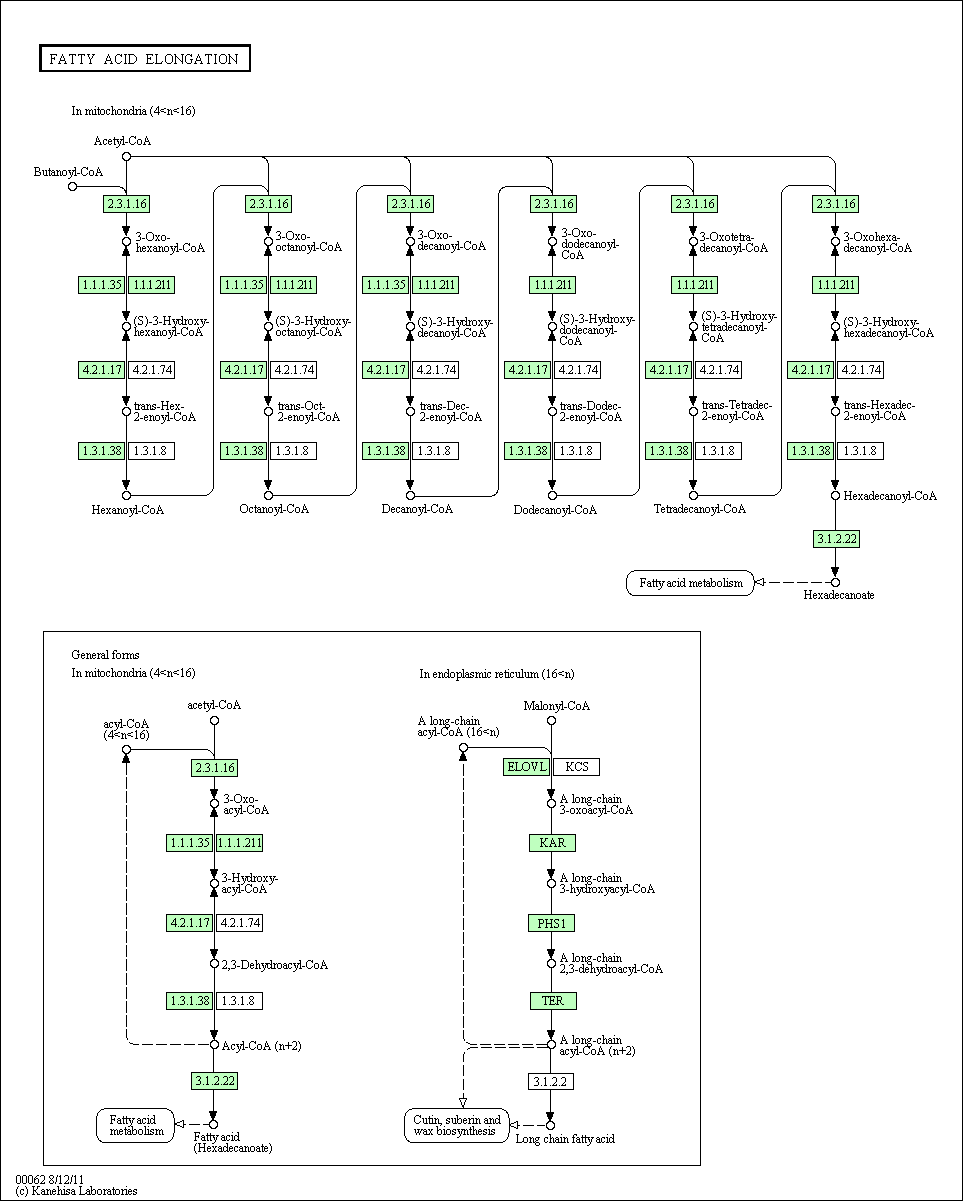 | 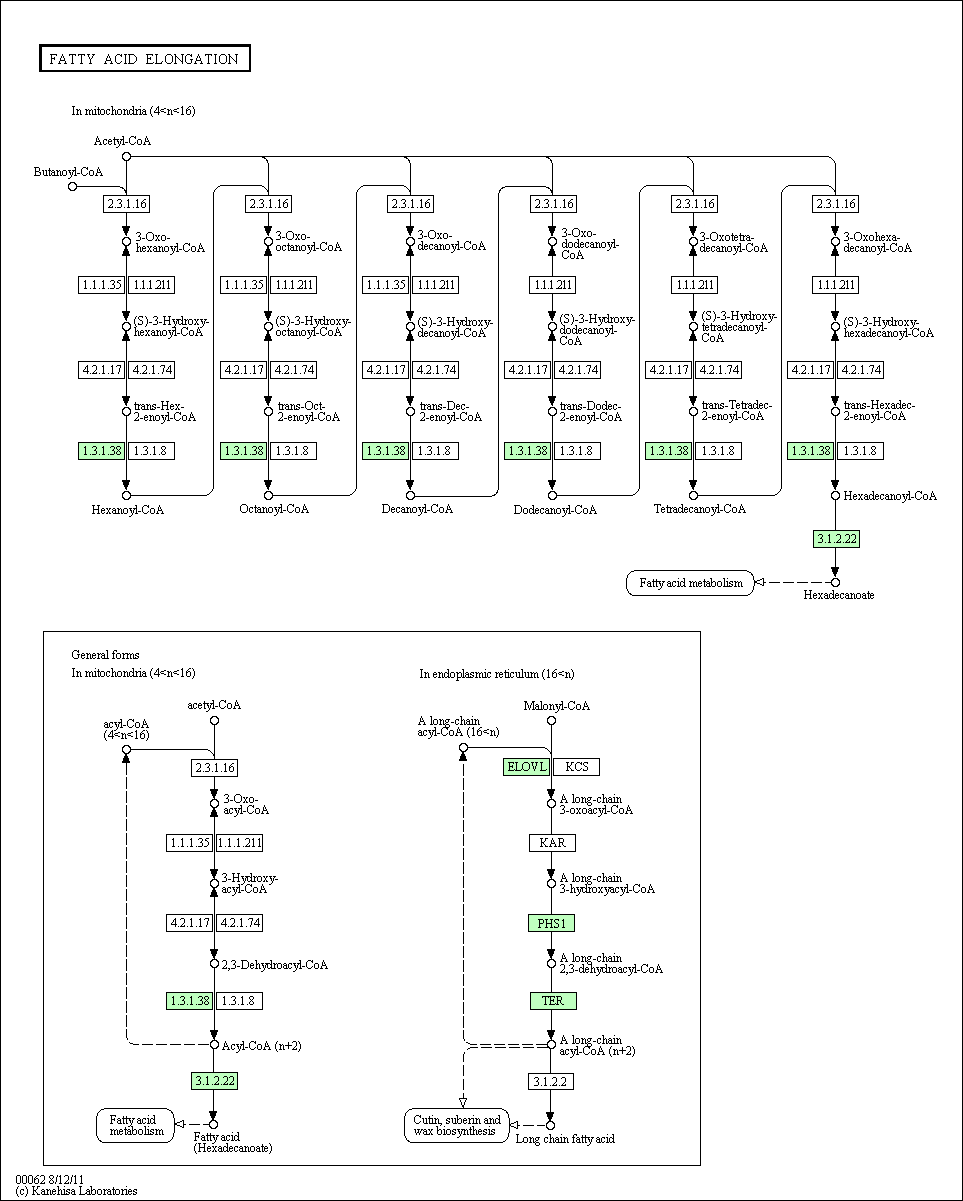 | 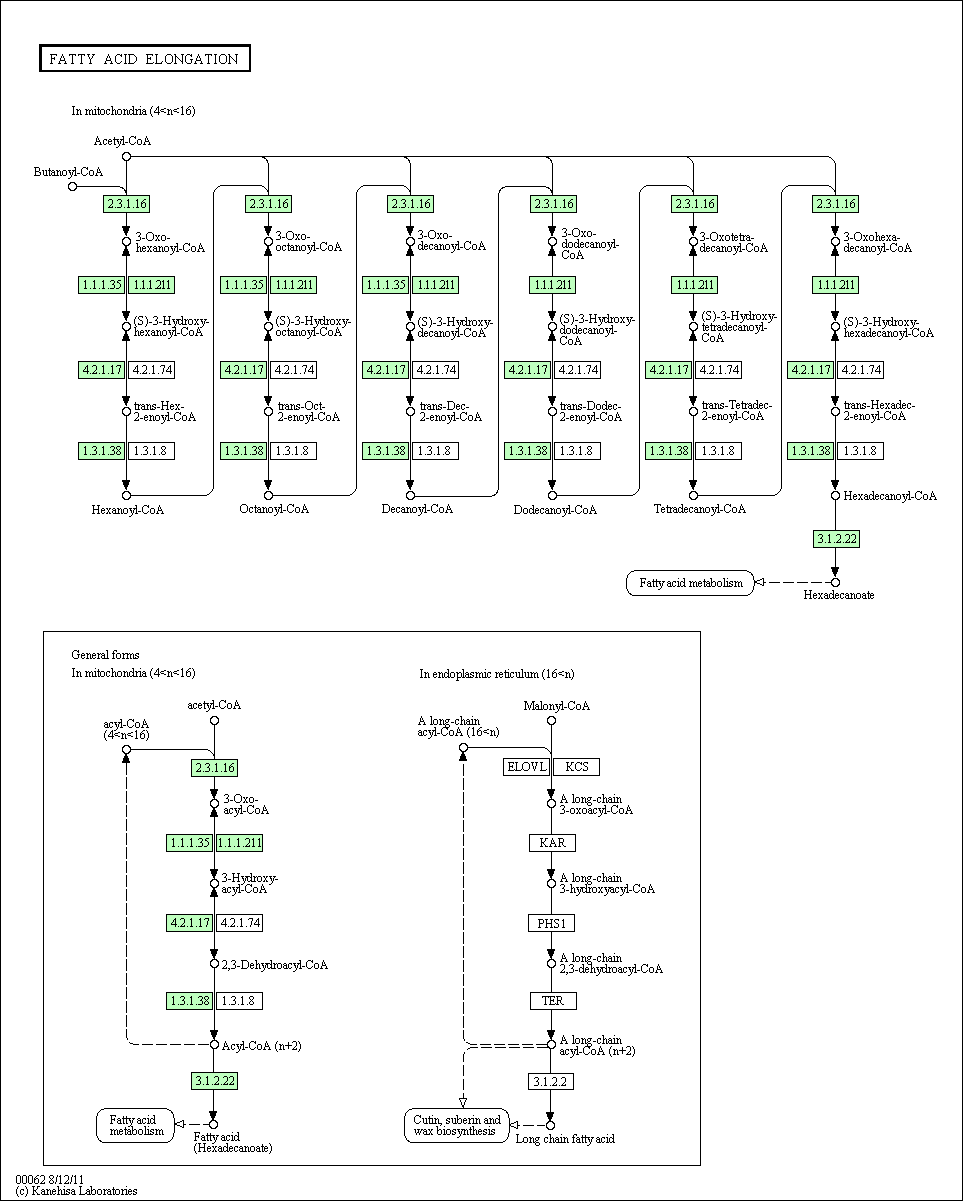 |
| Fatty acid biosynthesis | 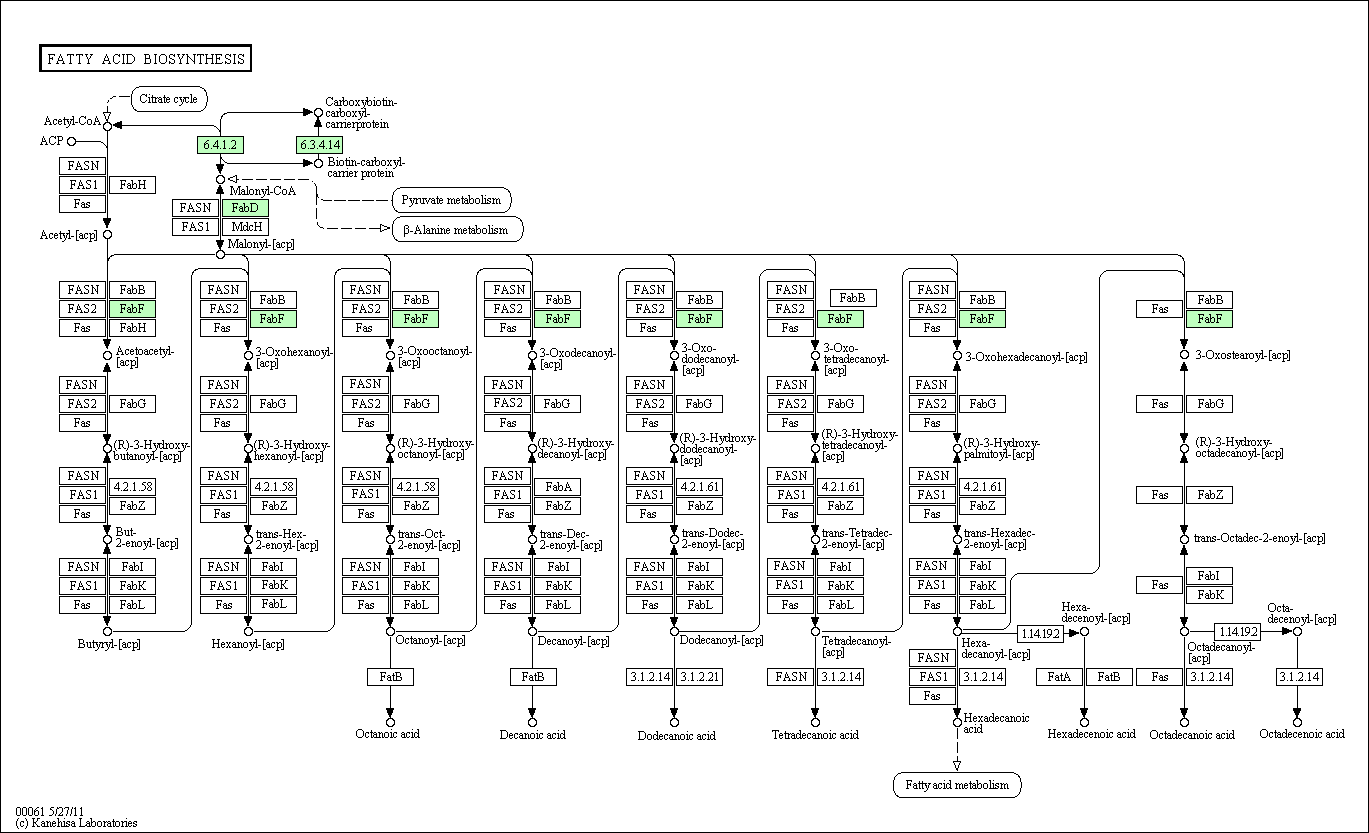 | 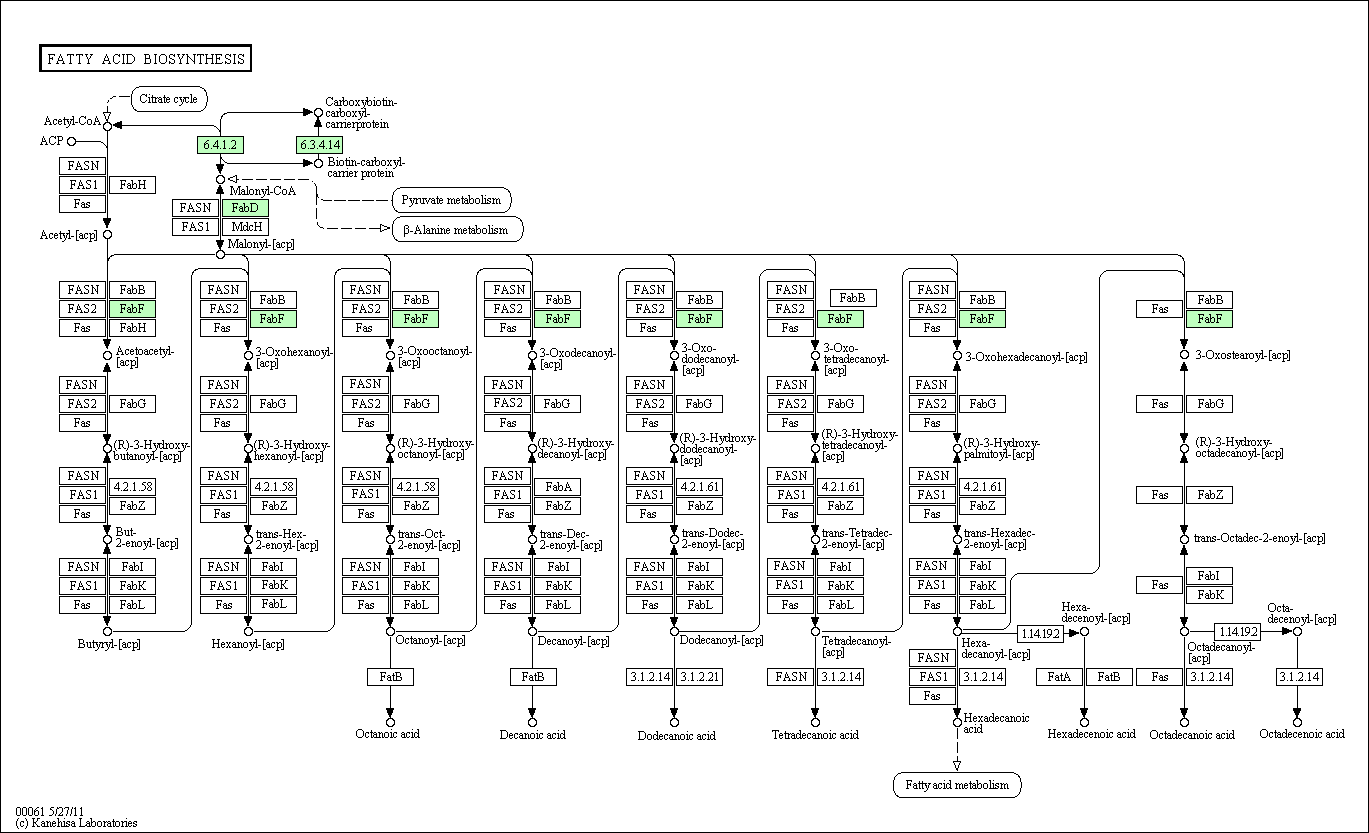 | 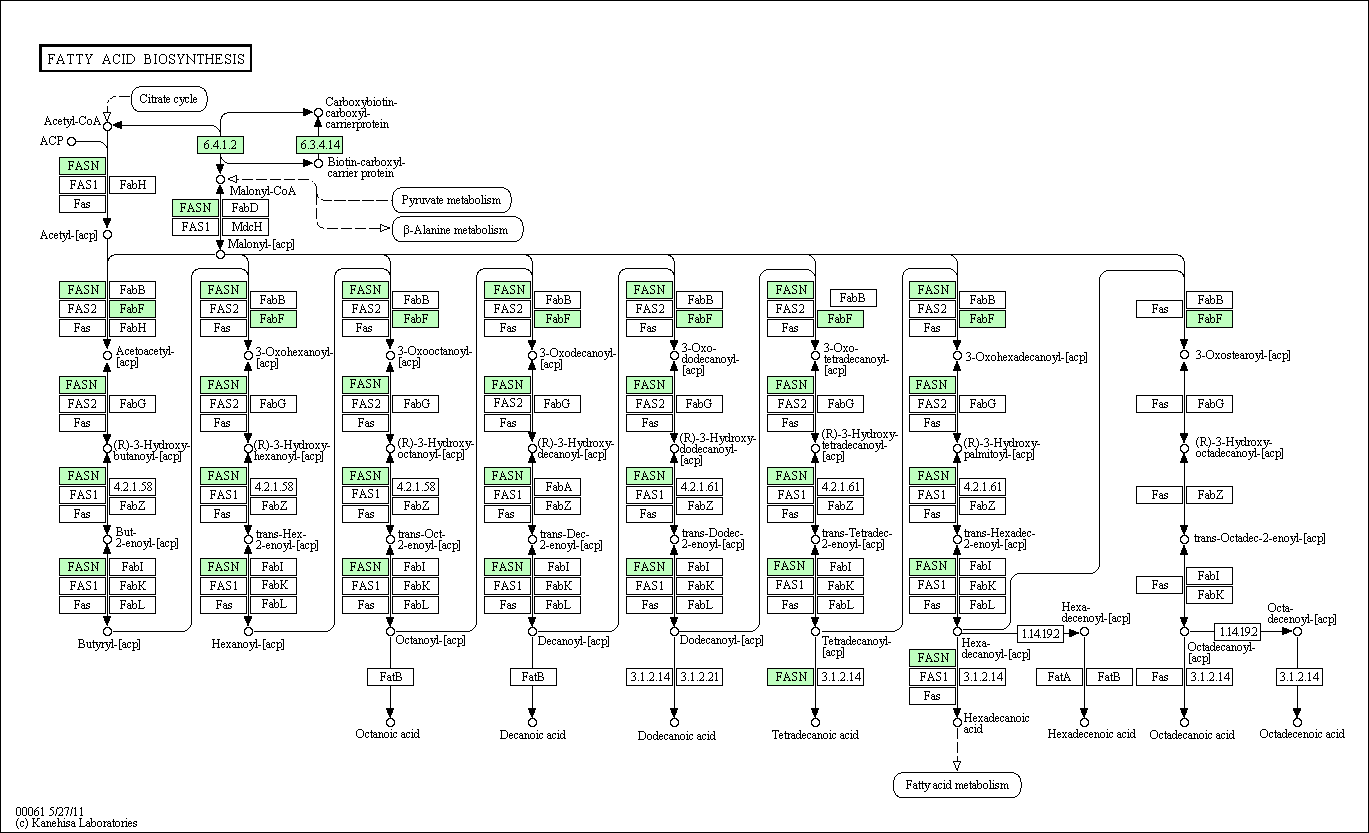 |
| Fatty acid metabolism | 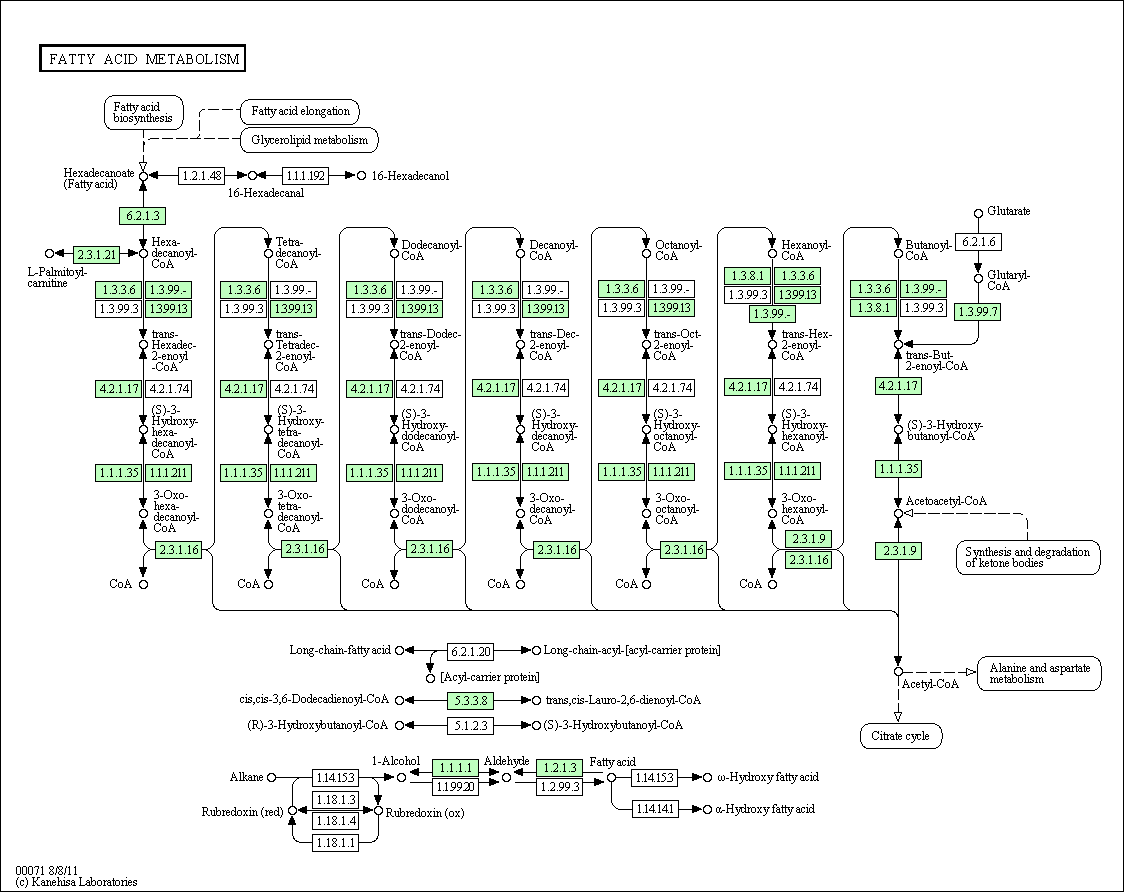 | 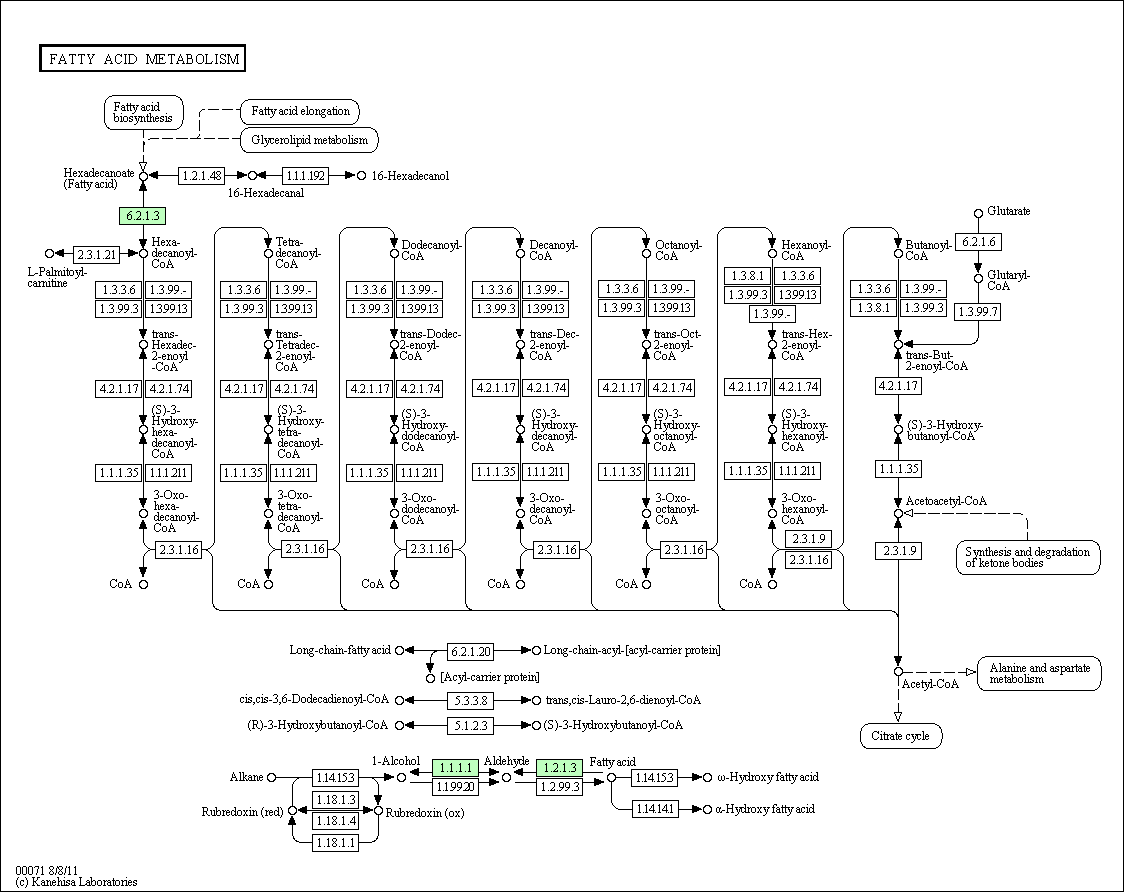 | 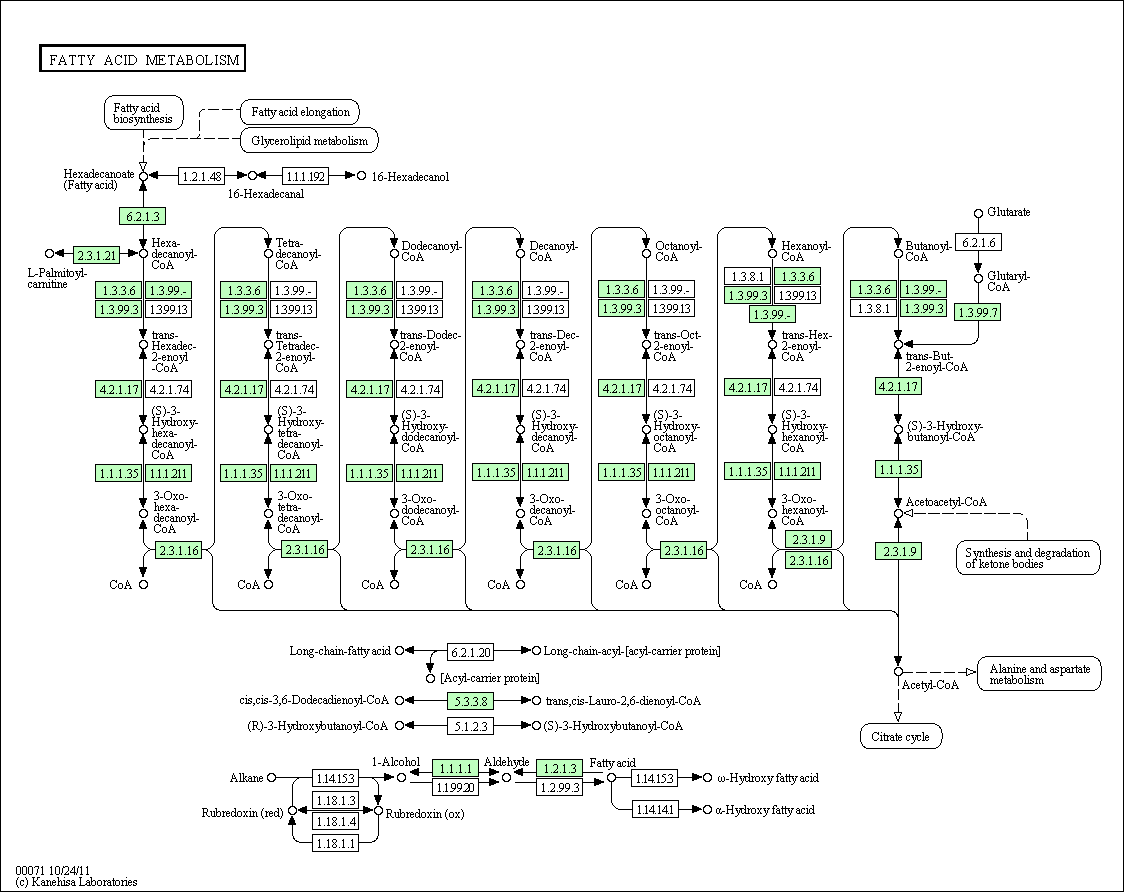 |

**Figure S5. Partial energy-related KEGG pathways of *C. sinensis*, *Schistosoma mansoni* and *Ascaris suum.***
