# Supplementary material for: The Carcinogenic Liver Fluke, Clonorchis sinensis: New Assembly, Reannotation and Analysis of the Genome and Characterization of Tissue Transcriptomes
Source: PLoS One. 2013 Jan 30;8(1):e54732. doi: 10.1371/journal.pone.0054732 (PMC3559784; doi:10.1371/journal.pone.0054732)
Supplement: Note S1 — Details of the upgraded genome. (DOC) [file pone.0054732.s021.doc]

**Detail of the upgraded genome**

**Genomic features of *C. sinensis***

Besides paired-end data previously published , we sequenced two paired-end and two mate-pair libraries again. In total, 263.38 million raw pairs of sequence reads (107X coverage) were produced，with 221.62 million (63X coverage) used for genome assembly after data filtering (Table S1). The assembled genome includes 4,348 scaffolds with a total length of 550 Mb. The upgraded version, with an N50 scaffold/contig length of more than 417/233 kb and the largest scaffold of more than 2 Mb, was improved compared with the previously published version (Table 1). The GC content was calculated to be approximately 43% (Figure S4). In *C. sinensis*, approximately 32% of the genome represents interspersed repeats, based on both known and *ab initio* repeat libraries (Figure S5). To estimate the gene coverage of the assembly, CEGMA core genes and assembled transcript data were mapped to the *C. sinensis* genome. As a result, approximately 97% of the transcripts were mapped, and on average, approximately 83% of the assembled transcripts were mapped to the same scaffold with at least 90% of their total length (Table S14).

Three methods, including similarity-based, *ab initio* methods and genome-guided assembly of RNA-Seq, were applied to identify protein-coding genes. After manual verification, a total of 13,634 gene models were retained as the final gene set. Detailed analysis of gene length, exon number per gene and gene density in *C. sinensis* showed similar patterns to those seen in both *S. japonicum* and *S. mansoni* (Table 2). Approximately 76.6% of the genes have homologs in the NCBI non-redundant database, and 58.8% can be classified using Gene Ontology terms. Overall, 79.6% of the genes could be annotated (Table S2).

In addition, non-coding RNAs were identified by the methods mentioned in our previous study . Nine rRNA fragments, 276 tRNAs, 482 small nucleolar RNAs, 149 small nuclear RNAs and 165 miRNA precursor genes were identified in the *C. sinensis* genome (Table S14), and 107 miRNAs had been found expressed previously (Table S15).

**Methods of assembly and annotation**

**DNA library construction and sequence analysis**

Besides paired-end data previously published , we sequenced two paired-end and two mate-pair libraries again. The two short-insert (400- and 550-bp) DNA libraries were constructed from the same fluke used in our previous study as described in the Paired-End Sample Preparation Guide (Illumina, San Diego, CA, USA). In addition, the two long-insert (2000- and 5000-bp) DNA libraries were constructed from twenty adult worms that were pooled together using the SOLiD Mate-Paired Library Construction Kit.

Cluster generation was performed on the cBot (Illumina), following the cBot User Guide. A paired-end sequencing run was then performed on the Genome Analyzer *IIx* (Illumina) as described in the Genome Analyzer *IIx* User Guide (Illumina). After masking adaptor sequences, removing contaminated reads and trimming low-quality reads, the cleaned data were processed for computational analysis.

**Genome assembly and repeat identification**

The whole genome shotgun sequencing raw data were filtered using Fastx-tools [[4](#_ENREF_4)][13] using the following criteria: 1) reads containing sequencing adaptors were removed; 2) reads were trimmed to 103bp for Celera Assembler limitation; 3) reads with low-quality (the percentage of nucletides with Q20 score were lower than 50%) were removed; and 4) artificial reads were removed.

The Celera Assembler v6.1 was used to assemble contigs and construct scaffolds with the mate-pair data using SSPACE . Finally, the gaps were filled with GapCloser [[7](#_ENREF_7)][16].

Known repetitive elements were identified using RepeatMasker with the Repbase database (version: 2009-06). A *de novo* repeat library was constructed using RepeatModeler , and default parameters were then used to generate consensus sequences and classification information for each repeat family. RepeatMasker was again run on the genome using the repeat library built with RepeatModeler.

**Gene prediction**

Predicted proteins from *S. japonicum* and *S. mansoni* were aligned to the *C. Sinensis* transcriptome to identify conserved genes. Because the GeneWise program is time consuming, proteins from *schistosome* were first aligned with the *C. sinensis* genome using genBlastA . Subsequently, matched genomic regions were extracted and GeneWise was used to identify exon/intron boundaries. The Program to Assemble Spliced Alignments (PASA) was used to generate spliced alignments of putative full-length cDNAs to the unmasked assembly, which was then used to train the *ab initio* gene prediction software, Augustus . Genscan was run using the model parameters for human. The RNA-Seq data from the four tissues were aligned to the genome using TopHat . Cufflinks was then used to assemble the transcripts with junction information. Gene predictions were generated using Augustus and Genscan, and spliced alignments of *S. japonicum* and *S. mansoni* proteins and transcripts produced by Cufflinks were integrated with EvidenceModeler . Transcript isoforms were constructed by Cufflinks with -g and default parameters, followed by Cuffcompare. Alternative splicing events were predicted using altSpliceFinder program in Ensembl API tools.

**Protein domain analysis**

InterProScan was run on all species (*C. sinensis*, *S. japonicum*, *S. mansoni*, *C. elegans*, *D. melanogaster*, *D. rerio*, *G. gallus* and *H. sapiens*) to predict protein sequences. Matched sequences tagged as ‘True Positive’ (status ‘T’) by InterProScan were retained.

**Functional annotation**

*C. sinensis* reference genes were mapped to KEGG pathways by BLAST (e-value<1e-5). BLAST searches against the Swiss-Prot database and NCBI non-redundant database (e-value<1e-5) were conducted to provide comprehensive functional annotation.

**CEGMA validation**

Using default parameters, the CEGMA set of 458 core eukaryotic genes was used to evaluate the completeness of the predicted gene models using the GenBlastA program.

**References**
